# Slower Calcium Handling Balances Faster Cross-Bridge Cycling in Human *MYBPC3* HCM

**DOI:** 10.1161/CIRCRESAHA.122.321956

**Published:** 2023-02-06

**Authors:** Josè Manuel Pioner, Giulia Vitale, Sonette Steczina, Marianna Langione, Francesca Margara, Lorenzo Santini, Francesco Giardini, Erica Lazzeri, Nicoletta Piroddi, Beatrice Scellini, Chiara Palandri, Maike Schuldt, Valentina Spinelli, Francesca Girolami, Francesco Mazzarotto, Jolanda van der Velden, Elisabetta Cerbai, Chiara Tesi, Iacopo Olivotto, Alfonso Bueno-Orovio, Leonardo Sacconi, Raffaele Coppini, Cecilia Ferrantini, Michael Regnier, Corrado Poggesi

**Affiliations:** Department of Clinical and Experimental Medicine, Division of Physiology (J.M.P., G.V., M.L., N.P., B.S., C.T., C.F., C. Poggesi), University of Florence, Italy.; Department of Biology (J.M.P.), University of Florence, Italy.; Department of NeuroFarBa (L. Santini, C. Palandri, V. Spinelli, E. Cerbai, R. Coppini), University of Florence, Italy.; European Laboratory for Non-Linear Spectroscopy (LENS) (F. Giardini, E. Lazzeri, C.F., C.P., E. Cerbai), University of Florence, Italy.; Department of Bioengineering, University of Washington, Seattle, WA (S.S., M.R.).; Department of Computer Science, University of Oxford, United Kingdom (F. Margara, A.B.-O.).; Amsterdam University Medical Center, Vrije Universiteit Amsterdam, Physiology, The Netherlands (M.S., J.v.d.V.).; Pediatric Cardiology (F. Girolami), IRCCS Meyer Children’s Hospital, Florence, Italy.; Cardiogenetics Unit (I.O.), IRCCS Meyer Children’s Hospital, Florence, Italy.; Department of Molecular and Translational Medicine, University of Brescia, Italy (F. Mazzarotto).; National Heart and Lung Institute, Imperial College London, London, United Kingdom (F. Mazzarotto).; Referral Center for Cardiomyopathies, Careggi University Hospital, Florence, Italy (I.O.).; Institute of Clinical Physiology (IFC), National Research Council, Florence, Italy (L. Sacconi).; Institute for Experimental Cardiovascular Medicine, Faculty of Medicine, University of Freiburg (L. Sacconi).

**Keywords:** founder effect, hypertrophic cardiomyopathy, myosin binding protein-C, personalized medicine, sarcomere energetics

## Abstract

The pathogenesis of *MYBPC3*-associated hypertrophic cardiomyopathy (HCM) is still unresolved. In our HCM patient cohort, a large and well-characterized population carrying the *MYBPC3*:c772G>A variant (p.Glu258Lys, E258K) provides the unique opportunity to study the basic mechanisms of *MYBPC3*-HCM with a comprehensive translational approach.

**Methods:**

We collected clinical and genetic data from 93 HCM patients carrying the *MYBPC3*:c772G>A variant. Functional perturbations were investigated using different biophysical techniques in left ventricular samples from 4 patients who underwent myectomy for refractory outflow obstruction, compared with samples from non-failing non-hypertrophic surgical patients and healthy donors. Human induced pluripotent stem cell (hiPSC)-derived cardiomyocytes and engineered heart tissues (EHTs) were also investigated.

**Results:**

Haplotype analysis revealed *MYBPC3*:c772G>A as a founder mutation in Tuscany. In ventricular myocardium, the mutation leads to reduced cMyBP-C (cardiac myosin binding protein-C) expression, supporting haploinsufficiency as the main primary disease mechanism. Mechanical studies in single myofibrils and permeabilized muscle strips highlighted faster cross-bridge cycling, and higher energy cost of tension generation. A novel approach based on tissue clearing and advanced optical microscopy supported the idea that the sarcomere energetics dysfunction is intrinsically related with the reduction in cMyBP-C. Studies in single cardiomyocytes (native and hiPSC-derived), intact trabeculae and hiPSC-EHTs revealed prolonged action potentials, slower Ca^2+^ transients and preserved twitch duration, suggesting that the slower excitation-contraction coupling counterbalanced the faster sarcomere kinetics. This conclusion was strengthened by in silico simulations.

**Conclusions:**

HCM-related *MYBPC3*:c772G>A mutation invariably impairs sarcomere energetics and cross-bridge cycling. Compensatory electrophysiological changes (eg, reduced potassium channel expression) appear to preserve twitch contraction parameters, but may expose patients to greater arrhythmic propensity and disease progression. Therapeutic approaches correcting the primary sarcomeric defects may prevent secondary cardiomyocyte remodeling.

**Novelty and Significance**

**What Is Known?**

- Mutations in the gene coding for the cMyBP-C (cardiac myosin-binding protein C) of the sarcomere (*MYPBC3* gene) are the most common variants associated with hypertrophic cardiomyopathy (HCM).
- Haploinsufficiency, that is, the reduction of the total amount of functional cMyBP-C protein within the sarcomeres, is generally regarded as the main mechanism determining primary myocardial dysfunction in patients carrying *MYBPC3* gene mutations.
- Adaptive/maladaptive changes of cardiomyocyte function (action potentials, ion channels, and intracellular calcium handling) have been reported in human HCM myocardium, albeit their relationship with the primary effects of different sarcomeric mutations remain largely unknown.

**What New Information Does This Article Contribute?**

- The c.772G>A mutation of *MYBPC3* is highly prevalent in the Tuscany region due to a founder effect.
- In the myofibrils of patients with the *MYBPC3*:c772G>A variant, cMyBP-C haploinsufficiency causes an acceleration of cross bridge kinetics in the sarcomeres and increased energetic cost of contraction, also supported by novel optical microscopy techniques.
- Electrophysiological changes (slower action potentials and prolonged calcium transients) appear to counterbalance the faster cross-bridge cycling, ultimately preserving the amplitude and duration of cardiac contraction, at the expense of cardiac electrical stability and diastolic function.

The presence of a large and well-characterized founder population of HCM patients carrying the *MYBPC3*:c772G>A variant offers the unique opportunity of studying the pathogenesis of HCM in multiple human samples where the disease is caused by the same mutation. The c.772G>A variant was studied here for the first time by comparing the functional properties of ventricular cardiomyocytes and trabeculae from human samples of surgical origin (myectomy operation) with myocytes and engineered muscles differentiated from human induced pluripotent stem cells (hiPSC) obtained from the same patients (hiPSC). Our results support the idea that, while the accelerated sarcomere kinetics would impair myocardial contraction, compensatory slowing of calcium handling preserves contraction parameters. Indeed, secondary changes of ion channels and calcium homeostasis were present in cardiomyocytes from myectomy samples and hiPSC, highlighting an early adaptive response to primary sarcomeric changes due to haploinsufficiency. As haploinsufficency is the primary mechanism of disease in nearly all HCM-linked *MYBPC3* mutations, our results could be extended to the whole population of *MYBPC3*-mutant subjects (20%–30% of all HCM patients). An early treatment of *MYBPC3*:c772G>A carriers with drugs that normalize cross-bridge kinetics and sarcomere energetics (eg, mavacamten) may prevent or reduce HCM-related pathology.

**Meet the First Author, see p 543**

Hypertrophic cardiomyopathy (HCM) is the most common inherited heart muscle disease, characterized by abnormal and asymmetric thickening of the left ventricle (LV), diastolic dysfunction, and structural remodeling of the myocardium (eg, myocyte/myofibril disarray). Most genotyped HCM patients harbor a mutation in one of the genes coding for cardiac sarcomeric proteins—hence, HCM is defined primarily as a disease of the sarcomere.^1^

A complex and unresolved chain of events leads from sarcomeric protein alterations to mechanical dysfunction and cardiac remodeling. We have previously shown that HCM associated mutations in both thick- and thin-filament proteins (ie, *MYH7*:p.R403Q and *TNNT*3:p.K280N),^2–4^ primarily increase cross-bridge cycling kinetics leading to increased energy consumption during tension generation (tension cost). Inefficient or excessive ATP utilization for tension development may play a central role in the pathogenesis of HCM.^5^ The energy depletion hypothesis is supported by several studies in HCM patients, human heart samples, and animal models.^2,3,6–8^

Mutations in *MYBPC3*, the gene coding for cMyBP-C (cardiac myosin-binding protein-C), represents the most common molecular etiology of HCM.^9^ cMyBP-C is a thick-filament-associated protein that localizes in the cross-bridge bearing C-zone of the A band within the cardiac sarcomere, where it plays important structural and functional roles.^10,11^ About 70% of known cMyBP-C mutations are either frameshift or nonsense variations, for which a haploinsufficiency mechanism has been postulated.^12,13^

Though less common than truncation mutations, a growing number of pathogenic missense mutations in *MYBPC3* have been identified in HCM.^14,15^ The c.772G>A has been repeatedly reported among *MYBPC3* variants, but its true nature is still debated.^16,17^ Although the c.772G>A transcript change is predicted to result in a missense substitution at position 258 (a lysine residue in place of glutamic acid, p.E258K), several lines of evidence indicate that this nucleotide change results in an exon skipping event—ultimately leading to a frame shift—by altering the last nucleotide of exon 6 and interfering with splicing.^18,19^ Further understanding of these chain of events may prove instrumental to understanding HCM pathogenesis and identifying novel therapeutic targets.

To date, the molecular mechanisms of the MYPBC3-c.772G>A pathogenicity remain controversial. By using different experimental models and approaches, we report that the *MYPBC3*-c.772G>A is a Tuscany founder mutation causing HCM. Our extensive set of analyses in HCM patient samples and human-induced pluripotent stem cell-derived cardiomyocytes (hiPSC-CMs) show that this founder mutation, most likely at early disease stages, causes (1) cMyBP-C deficiency, (2) impaired sarcomere energetics due to changes in cross-bridge cycling, and remodeling of excitation-contraction (E-C) coupling.

## Methods

### Data Availability

Data are available upon request.

An expanded Methods section is available in the Supplemental Material.

### Patient Population

The study included 93 HCM patients carrying the *MYBPC3:c*772G>A (p.Glu258Lys; E258K) mutation (Table 1). Clinical details for the 4 unrelated *MYBPC3*:c772G>A index patients (ID1, ID2, ID3 and ID4) who underwent surgical myectomy (providing cardiac samples for in vitro studies) are given in Table S1. The control cohorts for in vitro experiments were LV specimens from 11 nontransplanted donor hearts, 8 nonfailing nonhypertrophic surgical patients,^20^ and 2 healthy hiPSC lines. Clinical details for the 8 nonfailing nonhypertrophic surgical patients are given in Table S2. Written informed consent from each patient was obtained before surgery and the study was approved by the local Ethics Committee.

**Table 1.**
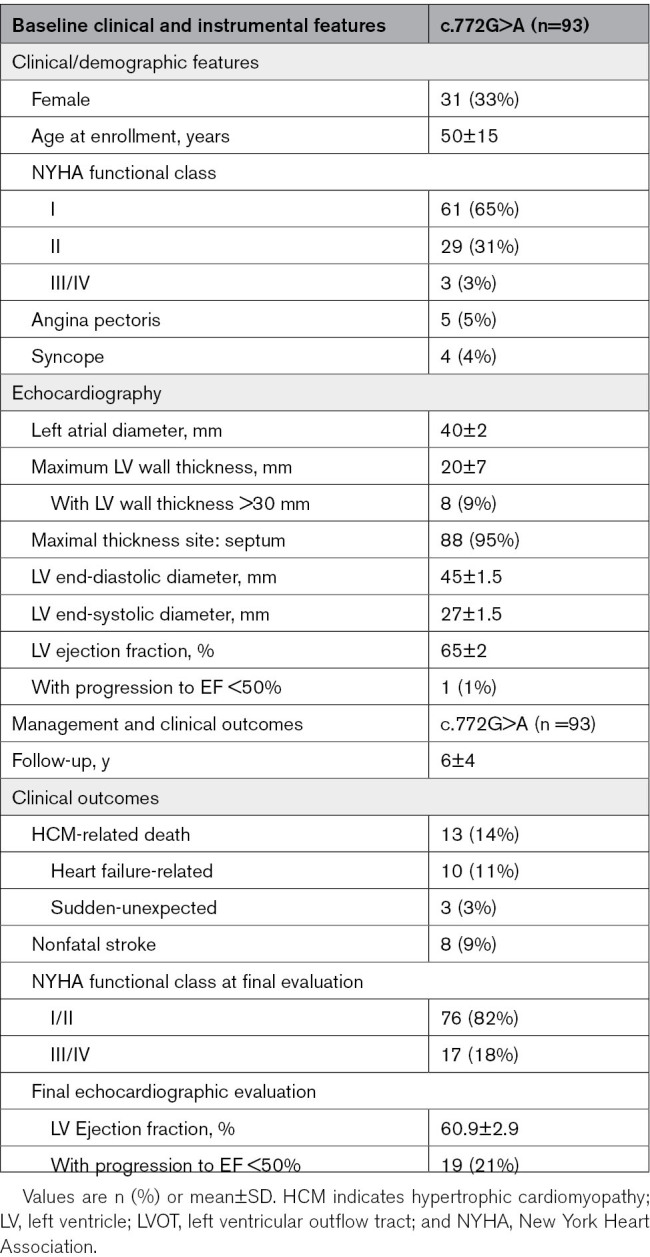
Clinical Features of *MYBCP3*-c.772G>A-HCM Patients

### Genetic Testing, Microsatellite, and Haplotype Analysis

Genetic analysis was performed by Sanger and Next Generation Sequencing (NGS). Segregation analysis was subsequently used to infer the individual haplotypes. Primers used for amplification and genotyping techniques are available in Table S3.

### Tissue Processing

After collection of surgical cardiac samples, trabeculae were cut for mechanical experiments, single cardiomyocytes isolated as previously described^21^ and the remaining tissue used for skinned strips, myofibril, and proteomic analysis.

### Tension Cost Measurements and 3-D Structure of Permeabilized Ventricular Strips

Sarcomere energetics was assessed in Triton permeabilized strips of mutant and donor ventricular samples.^6^ To exclude myocardial disarray as an artificial contributor to mechanical and energetic data, we employed a novel method able to quantify myocyte alignment, disarray, and contractile tissue content in tissue strips.^22^ The local cardiomyocyte’s orientation was detected with cellular resolution and represented as 3D vectors. Global Alignment quantifies the average alignment degree of the cells along the longitudinal axis of the preparation (Y), while local disarray quantifies the misalignment degree of nearby orientation vectors with respect to the mean local direction of the cells. The percentage of contractile tissue in the preparation is quantified as the ratio between the effective volume of labeled tissue (ie, total volume of cardiomyocytes thanks to the high-specificity of anti-α-actinin antibody staining) and the total volume of the preparation.

### Isolated Myofibrils

Single myofibrils were isolated from skinned mutant and donor ventricular samples and used for mechanical measurements.^2,3^

### Intact Cardiomyocytes and Trabeculae

On single cardiomyocytes isolated from mutant and control ventricular samples, perforated patch whole-cell current clamp was used to simultaneously monitor membrane potential and, [Ca^2+^]_i_ with the fluorescent calcium indicator Fluoforte. Intact ventricular trabeculae from the same ventricular samples were mounted on a force-recording apparatus to measure twitch contractions.^20^

### In Silico Simulations

Electromechanical simulations of human cardiomyocyte function in control and in the presence of the mutation were conducted in MATLAB using a previously validated computational framework.^23^

### Protein Analysis

Fast-frozen ventricular samples were processed to obtain total proteins, which were used for Western blot studies to assess expression as previously described.^13^ Septal specimens from 5 nontransplanted donor hearts were included as controls for Western blot. Uncropped blots are in Figure S7.

### Reverse Transcription–Polymerase Chain Reaction

mRNA isolated from septal specimens underwent reverse transcription, and the resulting cDNA was used for quantitative real-time polymerase chain reaction using predesigned assays for the following genes: *KCNQ1, KCNH2, KCNQ1, KNIP2*, and *KCNE*.

### Patient hiPSC Lines With Isogenic Corrected Cell Lines

Reprogrammed hiPSCs from ID3 (clone 1) was gene edited by CRISPR-Cas9 correction of c.772G>A confirming restoration of the normal genotype (corrected ID3; Figure S4). Single hiPSC-CMs were evaluated on micro-grooved substrates for cell shortening and simultaneous recording of calcium transients and action potential (AP) by fluorescent indicators (day 60 post differentiation). Engineered heart tissues (EHTs) were generated and mounted on a force-recording apparatus to measure twitch contractions. Whole cell protein analysis was performed at days 14-, 30-, 45-, and 60 post-differentiation to verify the expression of cMyBP-C during the maturation time.

### Statistical Analysis

In the figures, representative traces were selected based on the best possible approximation of the mean values calculated from each dataset; displayed images were chosen in order to closely reflect the average behavior or aspect of the represented group.

All group data are expressed as mean±SEM and the number of patients or number of hiPSC-CM differentiations (N) and the number of preparations (n) are indicated in the respective legends. Statistical analysis, taking into account non-Gaussian distribution, inequality of variances and within-subject correlation, was performed as detailed in the Online Expanded Methods. Differences between groups were considered significant when *P≤0.05 and **P≤0.01. For human sample data, the *P* values were calculated using linear-mixed models. For hiPSC data, we used 1-way ANOVA with a Tukey pot-hoc test.

### Study Approval

The study conforms to the principles of World Medical Association’s Declaration of Helsinki for medical research involving human subjects. Surgical cardiac and blood samples from patients were collected at the University of Florence with informed consent (protocol 2006/0024713, renewed May 2009 and protocol SILICO-FCM 777204 [04-15-2019]), approved by the Ethical Committee of Careggi Hospital. Written informed consent was received from all patients prior to participation.

## Results

### Characterization of the c.772G>A HCM Cohort: Demonstration of a Founder Effect in Tuscany

**Figure 1.**
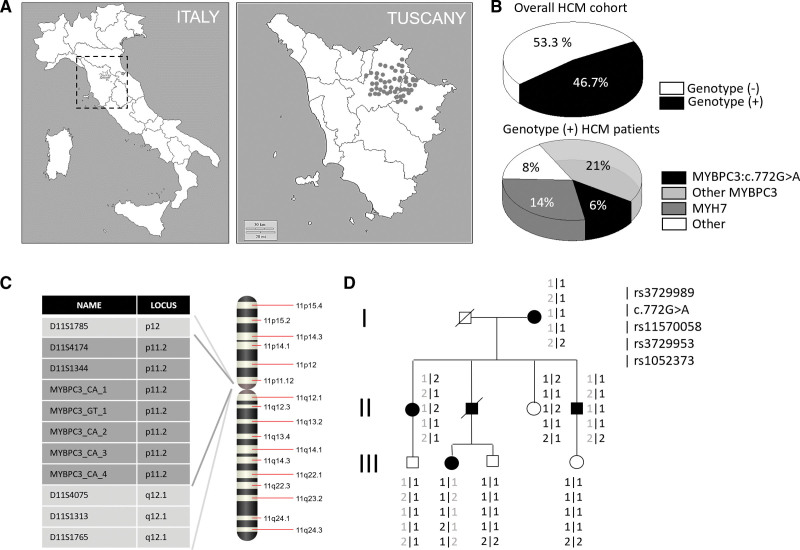
**Prevalence of the c.772G>A mutation in the Forence hypertrophic cardiomyopathy (HCM) cohort and demonstration of a founder effect. A**, Geographical origin of HCM patients carrying the c.772G>A variant. **B**, Prevalence of genotype-positive (obtained considering sarcomeric variants in *MYBPC3, MYH7, TNNT2, TNNI3, MYL2, MYL3, TPM1, or ACTC1* classified as pathogenic/likely pathogenic/of uncertain significance as per guidelines available at the time of testing) and genotype-negative patients among the overall HCM Florence cohort (top). Prevalence and distribution of sarcomeric myofilament protein gene mutations among the 46.7% genotype-positive HCM patients. **C** and **D**, PCR assays for 5 intragenic microsatellite markers (CA/GT dinucleotide repeats were designed, and their products sequenced to obtain genotypes for segregation analysis). Numbers were assigned randomly to repeat alleles, and haplotypes were reconstructed with Merlin. The haplotype marked in gray is shown to segregate consistently with HCM in one illustrative large pedigree (**D**).

Clinical diagnostic sequencing for HCM revealed the presence of the *MYBPC3*:c772G>A variant in 70 (5.8%) of the 1198 patients tested until November 2016.^24^ A further 23 carrier patients (comprising probands evaluated after November 2016 and/or family members) were included in our analysis. Allele-specific screening confirmed its absence in 129 healthy controls from the same geographical area. All 93 c.772G>A HCM patients originated from the North-Eastern part of Tuscany (Figure 1A). They represented 17% of genotype-positive individuals in the HCM Florence cohort (approximately one-third of all patients with *MYBPC3* mutations; Figure 1B). Haplotype analysis in 4 selected families revealed the presence of a unique haplotype spanning approximately 2 Megabases shared by all the c.772G>A individuals, confirming that the high prevalence of this otherwise rare variant is indeed due to a founder effect (Figure 1C and 1D).

At diagnosis, mean age of the 93 c.772G>A-HCM patients was 50±15 years (Table 1); 33% were women. Over 90% reported no or mild limiting symptoms (NYHA class I/II). Only 9% of patients were symptomatic due to angina (5%) or syncope (4%). The vast majority had classic localization of LV hypertrophy preferentially to the interventricular septum and anterior wall (94%); maximal LV wall thickness was 20±7 mm and LV ejection fraction (LVEF) at baseline was normal or supernormal except in one patient (Table 1).

**Figure 2.**
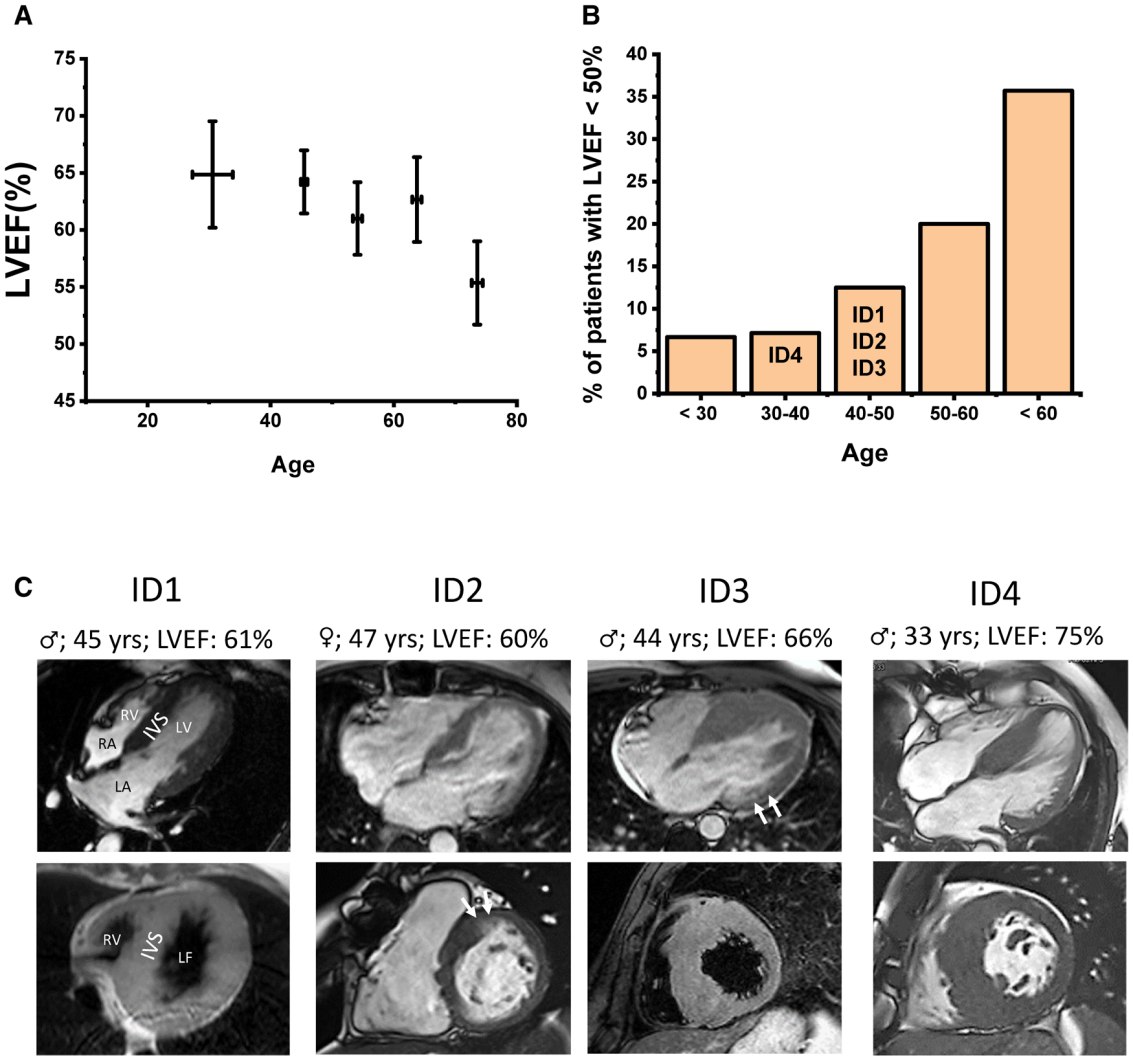
**Effect of age on left ventricular (LV) function in the c.772G>A population and cardiac magnetic resonance (CMR) imaging of the 4 patients selected for biophysical phenotyping. A**, Changes with age in the LV ejection fraction (LVEF; %) of the c.772G>A population. **B**, Percentage of c.772G>A patients with LVEF<50% in relation to age. **C**, CMR images from the 4 subjects who underwent myectomy from whom surgical samples were used for in vitro studies (sex, age, and LVEF on top of each image). Four chamber (Top) and short axis (bottom) views. Arrows show nonhomogeneous signal in the hypertrophic interventricular septum (IVS). LVEF is ≥60% in all 4 patients. Data collected at the last visit before myectomy. LA indicates left atrium; RA, right atrium; and RV, right ventricle.

During a mean follow-up of 6±4 years, 13 of the 93 patients (14%) died of cardiac causes and 8 (9%) experienced nonfatal cardioembolic strokes (Table 1); the rate of sudden cardiac death was 0.5%/year and heart failure-related death was 1.8%/year. Importantly, over one-fifth of the c.772G>A patients (n=19, 21%) showed advanced LV dysfunction at final evaluation (defined as LVEF <50% and/or restrictive diastolic pattern)—a proportion almost 3-fold that of the overall Florence cohort (*P*<0.01, unpublished observation) suggesting a considerable propensity to disease progression after age 45. As shown in Figure 2A and 2B, LVEF tended to decrease with age and the number of patients with LVEF<50% increased progressively after the fifth decade.

The 4 surgical patients who provided samples for in vitro studies were in the fourth (ID4: 33 years) and fifth decade (ID1, ID2, and ID3: 45, 47, and 44 years, respectively) and all had preserved ejection fraction (≥60%; Figure 2C; Table S1).

### The Energy Cost of Isometric Tension Is Increased in c.772G>A Sarcomeres

**Figure 3.**
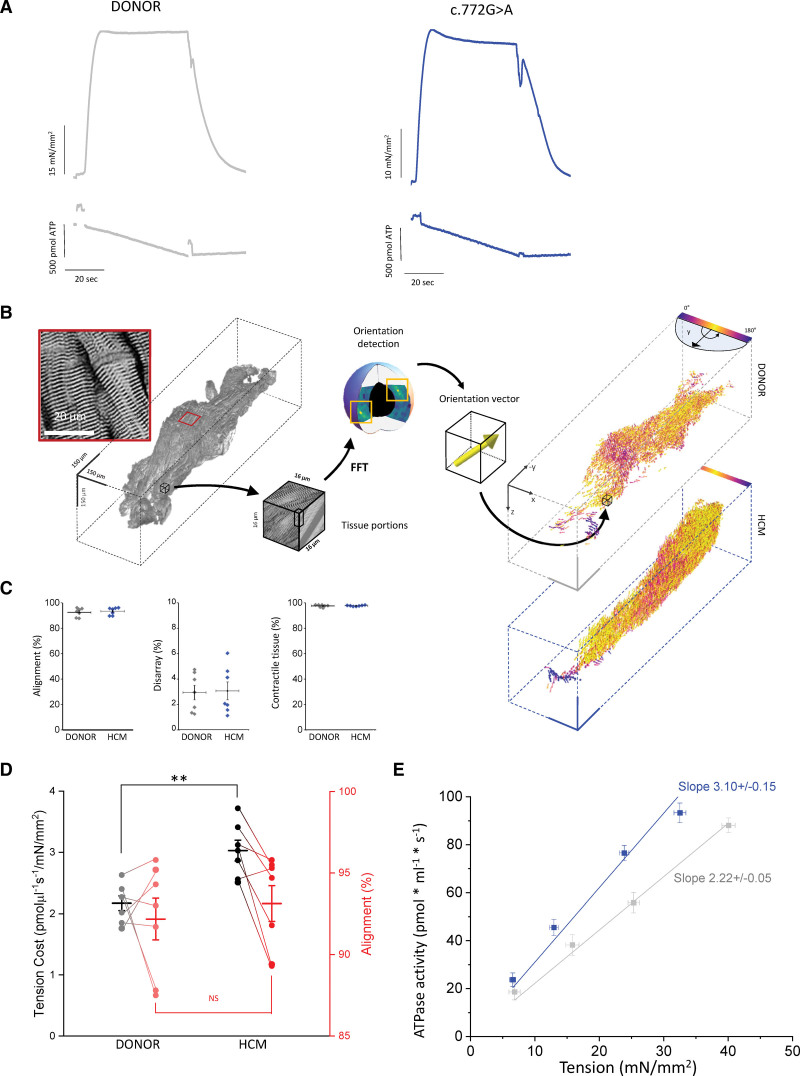
**Impact of the c.772G>A mutation on tension cost. A**, Representative isometric tension (top) and ATPase activity (bottom) recordings in skinned ventricular strips from donors and c.772G>A samples at saturating [Ca^2+^]. **B**, Three-dimensional reconstruction of a donor human muscle strip labeled with an anti-α-actinin antibody and imaged with a 2-photon fluorescence microscope. A magnification showing the Z-lines of the sarcomere at subcellular resolution is shown in the red square (pixel size: 0.44 μm). The local orientation of the contractile units is estimated by virtually dissecting the 3D reconstruction into 16 × 16 × 16 μm chunks. The entire cytoarchitecture is rearranged and represented in a vector space, where the color represents the degree of alignment with the principal axis of force production (Y). Two representative results for donor (gray box) and c.772G>A (blue box) muscle strips are shown. **C**, On the left, global alignment of contractile units (ie, the average component of the cellular orientations along the main traction axis (Y) of the preparation); the central graph shows disarray, expressed as the measure of the local angular dispersion of nearby cells; on the right, cardiomyocytes density, expressed as the ratio of the volume of contractile tissue vs total muscle volume. Donor N=3, n=7; c.772G>A N=3, n=7; donor and mutant values were compared using linear-mixed models; no significant differences were noted. **D**, One-to-one correlation between tension cost (in black and grey) and alignment (in red and light red) measured on donor and c.772G>A samples (donor N=3, n=7; c.772G>A N=3, n=7). Tension cost is measured as the ratio between maximal ATPase activity and maximal isometric force at saturating [Ca^2+^]. While a significant difference in tension cost was found between donor and hypertrophic cardiomyopathy (HCM) strips, no difference was found in cell alignment. *P* values calculated with linear mixed models; **P<0.01. **E**, Average pooled relations between active tension and rate of ATP consumption (tension cost is the slope of the relation) in c.772G>A (blue) and donor (gray) muscle strips (c.772G>A N=3, n=7; donor N=3, n=7). Data, expressed as mean±SEM, were obtained at different activating [Ca^2+^], pooled in 10%-wide steady-state tension bins and were fit by linear regression as indicated by the solid lines (slope values are shown in the panel). *The slopes of the 2 regression curves were compared using Student *t* test and found to be significantly different (*P*<0.05). **C–E**, N=number of patients, *n*=number of individual muscle strips. FFT indicates fast fourier transform.

To investigate the impact of the *MYBPC3*:c772G>A mutation on sarcomere energetics Triton-permeabilized ventricular strips from 3 patients and 2 control donors were used to simultaneously measure steady-state Ca^2+^-activated tension and ATPase activity under isometric conditions at 25 °C. Representative recordings of maximal isometric tension and ATPase activity at saturating Ca^2+^ concentration are shown in Figure 3A for both donor and mutant multicellular preparations. The comparison of mechanical and energetic measurements between donor and mutant ventricular strips may suffer from artifacts related to differences in the structural features of the 2 groups of preparations. Using a combination of advanced tissue clearing, labeling and optical imaging, we employed a method capable of reconstructing the whole ventricular strip with sufficient contrast and resolution to discriminate sarcomeric Z-lines across the whole length and thickness of the preparations (Figure 3B).^22^ A 3D Fast Fourier Transform-based cytoarchitecture analysis was applied to assess myofilament organization across the whole tissue volume. This imaging analysis allowed us to select strips with an adequate degree (near 100%) of cardiomyocyte alignment and density and a minimal degree (close to zero) of disarray (Figure 3C). By comparing structural and functional measurements one-to-one in a direct correlative manner, we found that HCM strips that had the same longitudinal cardiomyocyte alignment as the donor strips showed a significantly increased tension cost measured from the ratio between maximal ATP consumption and isometric tension at saturating [Ca^2+^] (Figure 3D; Figure S1).

Impaired energetics of the c.772G>A strips was confirmed when energy cost of isometric tension generation was calculated from the slope of the average relation between isometric steady-state tension and rate of ATP consumption measured at different levels of Ca^2+^-activation (Figure 3E). The slope of the tension-ATPase relation was significantly higher in the c.772G>A strips compared to the donor preparations.

### The c.772G>A Variant Accelerates the Kinetics of Myofibril Force Generation and Relaxation

To understand the mechanism responsible for the increased energy cost of tension generation in the c.772G>A myocardium, ventricular myofibrils from 3 c.772G>A patients and 5 donors were maximally Ca^2+^-activated (pCa4.5) and fully relaxed (pCa9) by rapid solution switching at 15 °C, as previously described.^3^

**Figure 4.**
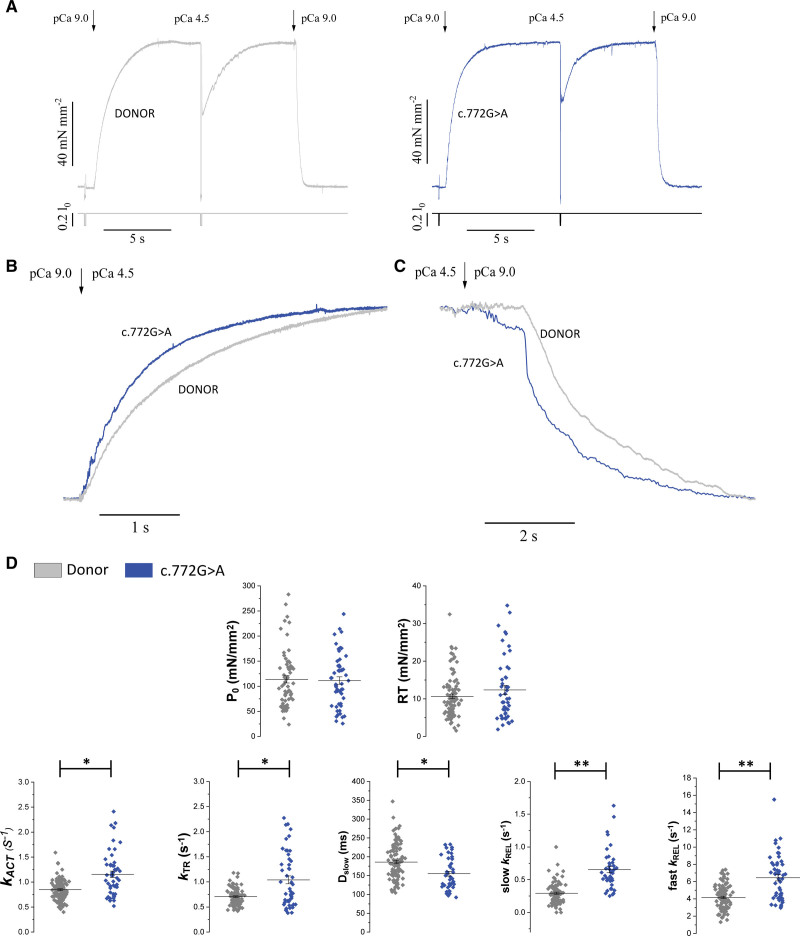
**Impact of *MYBPC3*:c772G>A mutation on myofibril mechanics and kinetics. A**, Representative tension responses (top traces) of donor (gray) and c.772G>A (blue) myofibrils maximally activated (pCa 4.5) and fully relaxed (pCa 9) by fast solution switching; the bottom traces are the lengths of the myofibrils that are subjected to release-restretch protocols at rest to measure resting tension and at steady-state contraction to measure *k*_TR_. **B** and **C**, The normalized tension curves from the c.772G>A and the donor myofibrils shown in **A** are superimposed to better compare the kinetics of force activation (**B**) and relaxation (**C**). **D**, Mechanical and kinetic parameters measured in donor (gray, N=5, n=100) and c.772G>A (blue, N=3, n=50) ventricular myofibrils. P_o_, maximal Ca^2+^-activated tension, RT, resting tension, *k*_ACT_, rate of tension activation, *k*_TR_, rate of tension redevelopment, slow *k*_REL_, rate of the initial slow isometric phase of relaxation, D_slow_, duration of the slow phase of relaxation, fast *k*_REL_, rate of the fast-exponential phase of relaxation. Lines and bars indicate mean±SEM. *P<0.05; **P<0.01. *P* values were calculated using linear mixed models. N=number of patients, n=number of myofibrils.

Figure 4A shows representative contraction-relaxation traces of both groups of myofibrils. Maximal Ca^2+^-activated tension was the same in c.772G>A myofibrils compared with donors while resting tension showed a trend to be higher in the mutant group though the difference was not significant (Figure 4D; Table S4). The rate of force activation, *k*_ACT_, and the rate of force redevelopment, *k*_TR_, were faster in c.772G>A myofibrils (Figure 4B through 4D; Table S4), indicating an accelerated apparent cross-bridge turnover in the mutant. Upon sudden Ca^2+^ removal, the time-course of myofibril force relaxation was biphasic, as previously described.^26,27^ The rate of the slow phase of relaxation (slow *k*_REL_) was markedly faster in the c.772G>A than in the donor myofibrils (Figure 4C and 4D; Table S4), indicating a faster apparent cross-bridge detachment rate under isometric conditions.^26,28–30^ The rate of fast relaxation (fast *k*_REL_) was also greater in the c.772G>A myofibrils (Figure 4C and 4D; Table S4). The duration of the slow force relaxation phase, D_slow_, is shorter in the mutant myofibrils (Figure 4D; Table S4).

The results (changes in kinetics with no changes in active tension) suggest that the apparent rates of cross-bridge attachment and detachment are both increased in the c.772G>A myofibrils by 1.5 to 2 times and that the change in the isometric detachment rate is responsible for the increased tension cost (1.5 to 2 times) measured in the skinned multicellular preparations.^30^

### Action Potentials and Ca^2+^ Transients are Prolonged in c.772G>A while Twitch Contraction and Response to Inotropic Stimuli are Preserved

**Figure 5.**
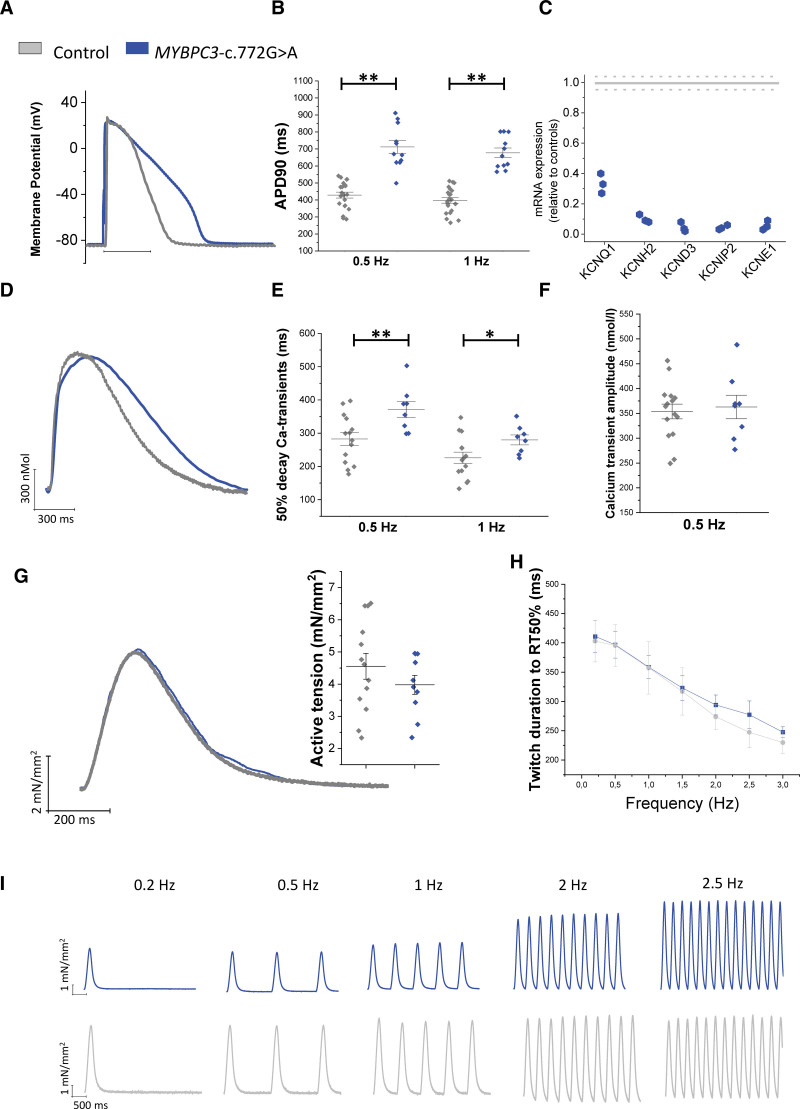
**Functional data from isolated cardiomyocytes and intact trabeculae. A**, Representative recording of action potential and (**B**) action potential duration at 90% repolarization (APD90%) from control cardiomyocytes (N=7, n=21) and c.772G>A cardiomyocytes (N=3, n=11), during stimulation at 0.5 and 1 Hz. **C**, RNA expression of potassium current genes in 3 hypertrophic cardiomyopathy (HCM) samples (relative to the 18S ribosomal RNA), expressed as a fraction of control values (N=1, indicated with a grey line with confidence intervals in dotted lines),: secondary I_Kr_ subunit (*KCNE2* or MIRP-1), I_Ks_ main channel gene (*KCNQ1* or KvLQT1) and I_Ks_ secondary subunit (*KCNE1* or minQ), potassium voltage-gated channel subfamily H member 2 (*KCNH2* or hERG1), Kv channel-interacting protein 2 (*KCNIP2*, KChIP2). **D**, Representative superimposed calcium transients from control and c.772G>A cardiomyocytes during stimulation at 1Hz. **E** and **F**, Ca^2+^ transient decay duration (time from peak to 50% decay) and amplitude from control cardiomyocytes (N=6, n=14) and c.772G>A cardiomyocytes (N=2, n=8), during stimulation at 0.5 and 1 Hz. **G**, Representative superimposed force twitches from control and c.772G>A intact trabeculae during stimulation at 0.5 and 1 Hz (inset: twitch amplitude at 0.5 Hz) and (**H** and **I**) twitch duration from stimulus to 50% of relaxation by increasing of stimulation frequency (0.2, 0.5, 1, 2, and 2.5 Hz). Controls: N=9; n=13^20^; c.772G>A: n=7, N=3. **E–H**, *P<0.05 and **P<0.01, calculated using linear mixed models. N=number of patients; and n=number of cells/trabeculae.

In isolated ventricular cardiomyocytes, the duration of action potentials (APs) and Ca^2+^ transients were markedly prolonged in the c.772G>A patients compared to controls (Figure 5A, 5B, 5D, 5E), with no changes in their amplitude (Figure 5F). Selective downregulation of the K^+^ channels at the transcriptional control level was indicated by the decreased expression of their main and regulatory subunits in HCM specimens (Figure 5C), suggesting a possible mechanism for the prolongation of action potentials, as previously reported.^20^

The contractile function of the c.772G>A ventricular myocardium was investigated using intact trabeculae dissected from the endocardial layer of myectomy specimens.^20^ As shown in Figure 5G, twitch amplitude was preserved in the c.772G>A trabeculae and its time-course was similar to that of controls (Figure 5H). Contractile reserve was also preserved in the HCM patient myocardium, as indicated by the positive inotropic response to high stimulation frequency (Figure 5I), stimulation pauses (Figure S3A), and beta-adrenergic stimulation (Figure S3B), similar to that measured in control myocardium. In addition, the c.772G>A ventricular muscle displayed a preserved adaptation of twitch duration to stimulation rate (Figure 5H and 5I), as observed previously in surgical samples of intact trabeculae from patients harboring different HCM causing mutations.^20^

The mechanism responsible for a preserved twitch duration with the underlying prolonged AP and calcium transients likely arises from the acceleration of cross-bridge cycling described above. To test this hypothesis, we employed a previously described model of HCM human cardiomyocytes^23,31^ (Table S5).

We report above that the c.772G>A mutation accelerates cross-bridge kinetics, as shown by the enhanced *k*_ACT_ and slow *k*_REL_. According to a simple 2-state cross-bridge model,^32^ under the present experimental conditions (nominally zero [P_i_]), *k*_ACT_ reports the sum of the apparent rate constants *f*_app_ and *g*_app_ that limits transition of cross-bridges from nonforce-generating to force-generating states and from force generating to nonforce-generating states, respectively. In terms of cross-bridge turnover kinetics, slow *k*_REL_ is a measure of *g*_app_, which in the model is also equal to the tension cost (isometric ATPase per unit force). Force is proportional to the apparent duty ratio [f*app*/(f*app*+g*app*)], and an increase in *g*_app_ should lead to a decline in the force generating capacity of the sarcomere unless the increase in *g*_app_ is compensated for by an increase in *f*_app_. The myofibril data support the idea that the c.772G>A mutation increases *f*_app_ and *g*_app_ to a similar extent, that is 1.5 to 2 folds. A similar increase in *g*_app_ was estimated from the change in tension cost.

**Figure 6.**
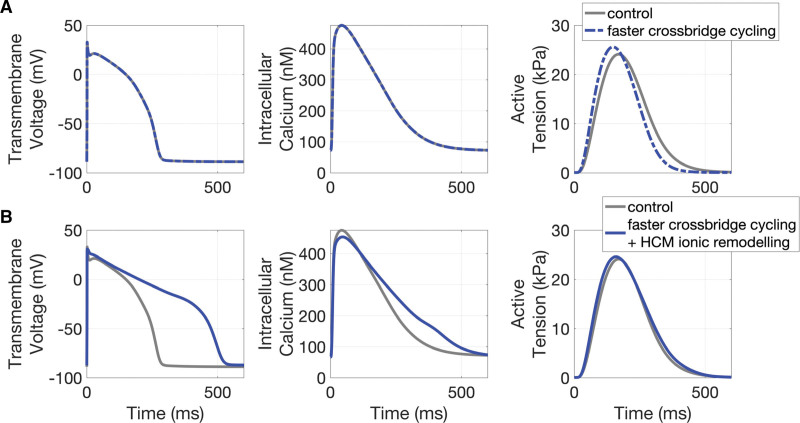
**Mechanistic simulations of human ventricular myocyte electromechanical function explain the impact of altered cross-bridge cycling on force generation, in the context of hypertrophic cardiomyopathy (HCM) ionic remodeling. A**, From left to right, comparison of action potential, calcium transient, and active tension waveforms in control (gray) and under faster cross-bridge cycling (blue). **B**, (from left to right) Comparison of action potential, calcium transient, and active tension waveforms in control (gray) and under faster cross-bridge cycling and HCM ionic remodeling (blue).

Figure 6A shows what happens if a 1.5- to 2-fold change of cross-bridges attachment and detachment rates is imposed in a control cardiomyocyte in the mathematical model (ie, with preserved AP and calcium transient). The resultant twitch contraction is accelerated (both peak and relaxation times) and slightly increased in amplitude. In Figure 6B, we mimic the prolongation of AP and calcium transient observed in c.772G>A and demonstrate that a 1.5- to 2-fold increase of cross-bridge attachment and detachment rates would in this case preserve the twitch duration. This provides proof of concept that the impact of increased cross-bridge cycling can be offset by slowing of the calcium transient.

### Analyses of cMyBP-C Protein Levels Unveil Haploinsufficiency in c.772G>A Myocardium and hiPSC-Derived Cardiomyocytes

**Figure 7.**
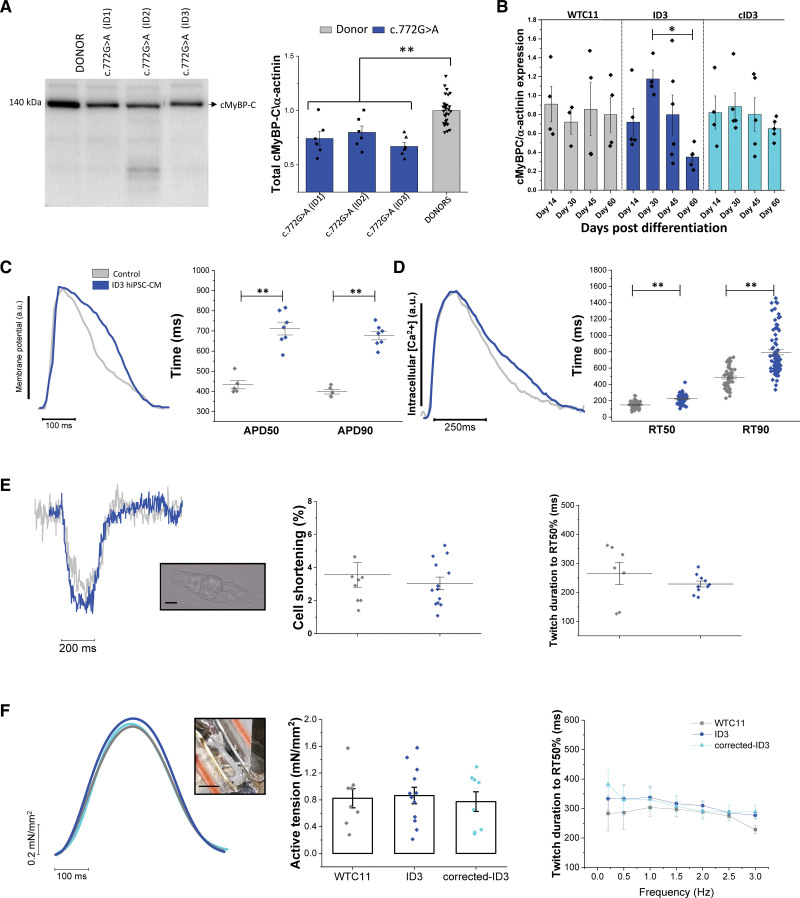
**Reduced cMyBP-C (cardiac myosin-binding protein-C) expression in myectomy samples and human-induced pluripotent stem cell-cardiomyocytes (hiPSC-CMs) from *MYBPC3*:c772G>A patients and impact of the mutation on the function of hiPSC-CMs and engineered heart tissues (EHTs). A**, The levels of full-length cMyBP-C were analyzed by Western Immunoblot with the use of the C2-14 antibody and normalized to α-actinin in each of the 4 c.772G>A myectomy samples (N=4; n=18) compared with donor myocardium (*N=*5; *n=*30). Loading of samples, staining, and phosphorylation analysis are reported in Figure S2 and uncropped blots in Figure S7. Statistical analysis was performed using linear mixed models; **P<0.01. **B**, Protein content was isolated from pellets of mutant (ID3) hiPSC-CMs and compared with its isogenic CRISPR-Cas9 corrected control (c.ID3) and the healthy control cell line (WTC11) at different maturation time points (day 14, 30, 45, and 60 postinitiation of differentiation). WTC11 N=7, n=1–5; ID3 N=8, n=1–4; c.ID3 N=7, *n*1–4; *P<0.05, 1-way ANOVA with Tukey test. **C**, Simultaneous recording of action potential and calcium transients by fluorescent indicators (Cal630 and FluoVolt, respectively). Single mutant hiPSC-CMs (ID3 N=4, n=178) were compared with a healthy control cell line (UC3-4, N=3, *n*=131) for action potential at 50% and 90% of duration (APD_50_, ms) and the duration from calcium transient peak to 50% and 90% of calcium transient decay (RT50, ms). **D**, Analysis of single cardiomyocyte contractility by video-edge detection; percentage of unloaded cell fractional shortening and twitch duration to 50% of relaxation were recorded at 1 Hz at 37 °C in Tyrode solution with 1.8 mM of extracellular calcium. N=3, n=8 for controls; N=3, n=13 for ID3. **F**, EHTs were measured in isometric conditions at 37 °C in the Krebs-Henselheit solution with 1.8 mM of [Ca^2+^] under imposed pacing. Scale bar=2 mm. Active tension and twitch duration to 50% of relaxation of patient EHTs (ID3 N=7, n=12) and compared to thecorrected isogenic control (c.ID3 N=5, n=7) and the healthy control (WTC11 N=3, n=8). C–F, N=number of differentiations, n=number of replicates. One-way ANOVA with a Tukey post-hoc test was used to assess differences between the groups; *P<0.05 and **P<0.01 versus controls (WTC11 or UC3-4).

The molecular pathomechanism underlying HCM associated with *MYBPC3* mutations has often been identified in cMyBP-C protein deficiency. To test if the same mechanism is associated with the *MYBPC3*:c772G>A mutation, we analyzed cMyBP-C protein expression in the myectomy samples used for in vitro experiments. The levels of full-length cMyBP-C (relative to α-actinin) were ~30% lower in each of the 3 c.772G>A samples compared with donor myocardium (Figure 7A; Figure S7). Further analyses of cMyBP-C protein level and phosphorylation at specific phospho-sites are reported in Figures S2 and S7.

The levels of full-length cMyBP-C protein were also analyzed in cardiomyocytes derived from the isogenic pair of patient hiPSC cell lines (ID3 and corrected ID3) and from the healthy WTC11 cell line between day 14 and 60 after initiation of differentiation. The Western immunoblot normalized to sarcomeric α-actinin confirmed that the relative expression of cMyBP-C was reduced in the mutant compared to the healthy cardiomyocytes and restored in the isogenic corrected controls from the patient (Figure 7B; Figure S5). This result supports the haploinsufficiency hypothesis prediction predicated on the results from myectomy samples.

### hiPSC-Derived Cardiomyocytes and EHTs Recapitulate the Biophysical Phenotype Observed in Patients’ Samples

In individual hiPSC-CMs we used an optical method for the simultaneous measurement of AP and calcium transients with fluorescent indicators at day 60 post-differentiation.^33^ As shown in Figure 7C and 7D, the duration of the AP and calcium transients were slower in the ID3 patient hiPSC-CMs compared with controls. These findings are consistent with the AP and calcium transients that were observed from cardiomyocytes that had been freshly isolated from the patient surgical samples. Compared to controls, ID3 patient hiPSC-CMs had similar fractional shortening, indicating preserved cell contractility (Figure 7E). Moreover, the time course of the twitch was comparable to that of the healthy cell line (Figure 7E).

Finally, to more closely recapitulate tissue level conditions, EHTs were fabricated and followed until day 50 while measuring auxotonic spontaneous contraction and frequency (Figure S6). ID3-EHTs were assessed for steady-state isometric twitch tension and kinetics at day 50 after differentiation, under imposed pacing rates. Compared to the CRISPR-Cas9 corrected ID3- and WTC11-EHTs, ID3-EHTs showed preserved twitch amplitude together with similar contraction time-course (both force rise and relaxation) and rate adaptation to twitch duration (Figure 7F). Engineered heart tissue contractile reserve was also preserved, as indicated by the positive inotropic response to stimulation pauses (Figure S3C and S3D) and high extracellular calcium ([Ca^2+^]_out_ up to 4 mM; Figure S3E and S3F).

## Discussion

Since the 1990s, the genetic heterogeneity of HCM has been well established,^34^ and a correspondingly large clinical variability has limited the interpretation and clinical actionability of genotype-phenotype correlation studies. The heterozygous variant c.772G>A in the *MYBPC3* gene was a substantial subset of 93 individuals (or 7.8%) of the Florence HCM patient cohort, all of them originating from the north-eastern part of Tuscany, allowing detailed insight into the pathophysiology of this mutation. Notably, c.772G>A is characterized by peculiar clinical features including marked propensity to systolic dysfunction and disease progression (occurring in 1 patient out of 5).^35,36^ The c.772G>A mutation is considered a rare variant worldwide, with an allele frequency <1% in the Exome Aggregation Consortium (ExAC) dataset (http://exac.broadinstitute.org/). Haplotype analysis confirmed that the locally high prevalence of this variant is indeed due to a founder effect.

We sought to address the molecular mechanism through which the c.772G>A variant is pathogenic. Western blot analysis showed a reduction in full-length cMyBP-C levels (~30%) in all 3 c.772G>A myectomy samples, compared to donor tissue (Figure 7A). In hiPSC-CMs from the ID3 patient, the protein expression of cMyBP-C (relative to α-actinin) was decreased in later stages (60 days after initiation of differentiation), but protein levels were preserved in a CRISPR-Cas9-corrected isogenic cell line (Figure 7B). This suggests that the mutation may be the cause of the reduced expression of cMyBP-C in mutant hearts, further supporting the hypothesis of protein haploinsufficiency as the main pathophysiological mechanism. Several studies suggested that truncating mutations in cMyBP-C diminish total protein level and cause HCM through haploinsufficiency.^12,13,25,37^ The c.772G>A variant affects the splice site consensus sequence at the last nucleotide of exon 6 in the *MYBPC3* coding sequence, leading to an out-of-frame skipping of exon 6 in the *MYBPC3* gene.^25,38^ In contrast to functional studies on murine engineered cardiac tissue carrying the same mutation, where a missense-mutated (p.Glu258Lys, E258K) or a truncated cMyBP-C was observed to be incorporated into cardiac sarcomeres and directly impaired contractile function,^16^ the expression of a truncated protein was never detected in affected heart tissue from HCM patients carrying the c.772G>A variant.^12,37^ The c.772G>A is the last base of exon 6 which results in splicing out of exon 6, causing a frameshift and then downstream termination codon. The mRNA is not truncated, but contains a premature termination codon, which makes it susceptible to nonsense-mediated decay. Previous studies indicated the E258K as a splice site mutation and the missense protein has never been shown.^38^ Another possible mechanism associated with haploinsufficiency is supported by other findings using heterozygous *MYBPC3*-mutant hiPSC-CMs, which reported reduced cMyBP-C synthesis rates, partially counteracted by a slower rate of cMyBP-C degradation.^39^

We analyzed the functional consequences of the mutation at the myofilament level and its impact on sarcomere energetics. Isolated myofibrils had markedly enhanced kinetics of force development and relaxation (kACT and slow kREL, respectively), but no change of myofibril maximal force. Given that slow *k*_REL_≈g_app_,^30^ the observed increase in cross-bridge detachment in c.772G>A myofibrils predicts an increase in the amount of ATP spent to generate a given amount of isometric force (ie, tension cost). Indeed, we identified higher tension cost in demembranated cardiac muscle strips isolated from c.772G>A myectomy samples compared to donor patients. Faster cross-bridge detachment under isometric conditions and higher energy cost of tension generation are common features of several HCM-associated mutations.^2,3,8,28^ Notably, the possibility that less efficient force generation was caused by fiber misalignment/disarray was excluded, thanks to a newly developed structural correlative approach featuring reconstruction of the whole 3D structure of each ventricular strip.^22^ Thus, we speculate that the increased tension cost is a direct consequence of the c.772G>A mutation. While a marked impact of many *MYH7* or *TNNT2* HCM variants on cross-bridge kinetics and sarcomere energetics is expected,^2–4^ the effects of *MYBPC3* mutations are less straightforward. Our results support the idea that haploinsufficiency is primarily responsible for sarcomere functional abnormalities due to this particular *MYBPC3* mutation. Normally, the N-terminal C1C2 region of cMyBP-C interacts with myosin heads in the S2 region, inhibiting its binding to actin and thus, reducing cross-bridge formation and cycling.^40,41^ Reduced cMyBP-C levels likely diminish this inhibitory interaction,^42,43^ consistent with the observed acceleration of cross-bridge cycling rates and the increased ATP consumption for tension development.

In line with previous observations in cardiomyocytes isolated from HCM patients with different genetic backgrounds, c.772G>A-cardiomyocytes showed prolonged APs and slower Ca^2+^ transients.^20^ A reduction of the expression of K^+^ repolarizing channels, as observed by reduced mRNA levels can at least partially account for AP prolongation. These acquired electrophysiological changes may represent universal maladaptive mechanisms that are common to all forms of HCM remodeling, regardless of genotype.^6,20^ In the case of the c.772G>A mutation, we verified by in silico simulations that the prolonged duration of APs is sufficient to prolong calcium transients and counterbalance the faster rate of cross-bridge turnover. Changes of the expression and phosphorylation levels of other E-C coupling proteins in HCM cardiomyocytes (decreased SERCA expression, increased Ca-calmodulin protein kinase II -CaMKII- activity) may also have contributed to the prolongation of Ca^2+^ transients, as previously observed in human HCM samples carrying different mutations.^20,44^ In the absence of AP and Ca^2+^ transient prolongation, twitch duration would be shortened. Thus, the simulations support the idea that the presence of concurrent prolongation of APs and Ca^2+^ transients is the reason why the time course and amplitude of contraction in c.772G>A myocardium are similar to those of controls, despite the faster cross-bridge turnover. This interpretation suggests that electrophysiological changes are an essential adaptation of c.772G>A myocardium aimed at counteracting the effects of the faster cross-bridge turnover, at the expense of cellular electrical stability. Indeed, prolongation of APs and Ca^2+^ transients is associated with the development of early and delayed afterdepolarizations.^6,20^ In this regard, electrophysiological changes are likely to appear early during disease development,^45^ in the prehypertrophic stages of the disease. The results obtained from hiPSC lines are in support of this hypothesis. It is generally accepted that hiPSC-derived cardiomyocytes mimic cells in their developmental stage^46^ and are therefore representative of the early, prehypertrophic stage of HCM typical of young mutation carriers. Electrophysiological and E-C coupling remodeling was present in hiPSC-CMs from c.772G>A patients, suggesting that this may be an initial adaptative mechanism that starts even before the onset of overt hypertrophy. Nonetheless, electrophysiological changes at the cardiomyocyte level might expose patients to an increased arrhythmogenic risk even at this early stage of disease. It is important to note that the molecular mechanisms linking impaired sarcomere function to E-C coupling remodeling are still unknown and need to be addressed. This, however, is far beyond the scopes of the present work and would require dedicated molecular approaches.

These results also support the idea that an early treatment of *MYBPC3*:c772G>A carrier may prevent or reduce HCM-related pathology, for instance, using the novel myosin inhibitors currently employed in clinical trials on obstructive and nonobstructive HCM patients. cMyBP-C has been suggested to regulate cardiac function by modulating and maintaining the “super-relaxed” state of myosin.^47,48^ The decreased super-relaxed fraction results in an increased number of myosin heads capable of interacting with actin, leading to higher sarcomeric energetic requirements, both at rest and during force development. Specifically, mavacamten^49^ may act by stabilizing the super-relaxed state in cardiac muscle,^50,51^ countering myocardial hyper-contractility, thus normalizing myocardial energetics.^52^ In addition, our study is consistent with the hypothesis that targeting early electrophysiological and E-C coupling changes may exert disease modifying effects, as well as reducing the risk of arrhythmias. Indeed, late Na^+^ current blockers such as ranolazine or disopyramide acutely reduced cellular arrhythmias in human myocardium and prevented disease progression during long-term treatment in HCM mouse models.^20,45,53,54^ Similarly, treatment of young prehypertrophic mutation-carrier patients with the calcium channel blocker diltiazem slowed the development of HCM.^55^ As cardiomyocyte calcium overload and the increased activity of CaMKII (calmodulin kinase) are hallmarks of sarcomeric HCM and drive disease progression,^20,44,56^ halting the early E-C-coupling changes with targeted therapies may be as effective as directly addressing the dysfunctional sarcomeres, with the added benefit of protecting against cellular arrhythmias.

## Article Information

### Acknowledgments

The authors thank Max Goebel (University of Amsterdam) for Western Blotting analysis; Alessia Tomberli (Cardiomyopathy Center, Careggi Hospital) for patient recruitment and blood sampling.

### Author Contributions

All authors contributed to the overall study design, interpretation of the results, writing of the manuscript and approved the final version of this manuscript. In details, G. Vitale, J.M. Pioner, C. Tesi, C. Ferrantini, C. Poggesi, and R. Coppini contributed to the collection and analysis of the intact and skinned preparations. J.M. Pioner, G. Vitale, N. Piroddi, B. Scellini, C. Ferrantini, C. Tesi, and C. Poggesi contributed to the collection and analysis of myofibril data. M. Schuldt and J. van der Velden contributed to the myectomy protein expression data. F. Mazzarotto and F. Girolami contributed to the genetic analysis. I. Olivotto, R. Coppini, C. Ferrantini, C. Poggesi provide patient and clinical data. F. Giardini, E. Lazzeri, and L. Sacconi contributed to the design of the method for structural analysis. J.M. Pioner, S. Steczina, M. Langione, L. Santini, C. Palandri, R. Coppini, M. Regnier, F. Mazzarotto, and E. Cerbai contributed to the generation and analysis of hiPSC experiments. F. Margara and A. Bueno-Orovio performed and analyzed the in silico simulation.

### Sources of Funding

This project has received funding from the European Union’s Horizon 2020 under grant agreement No. 777204 SILICOFCM (C. Poggesi, M. Regnier, I. Olivotto) and under grant agreement No 952166 REPAIR (C. Poggesi, E. Cerbai, L. Sacconi, C. Ferrantini), from the Netherlands Organization for Sciences (NWO)-ZonMW (VICI 91818602), the Dutch Heart Foundation CVON/DCVA DOUBLE DOSE 2020B005 (M. Schuldt), NWO Human Models grant Proper Therapy (No. 18953) and British Heart Foundation FS/17/22/32644 (A. Bueno-Orovio). NIH grant RM1 GM131981 (M. Regnier). This research used resources of the University of Washington Center for Translational Muscle Research, supported by the NIH National Institute of Arthritis and Musculoskeletal and Skin D iseases under Award Number P30AR07 4990 (M. Regnier).

### Disclosures

None.

### Supplemental Material

Expanded Materials and Methods

Tables S1–S5

Figures S1–S7

Examples of statistical analysis

## Supplementary Material


