# Supplementary material for: Slower Calcium Handling Balances Faster Cross-Bridge Cycling in Human MYBPC3 HCM
Source: Circ Res. 2023 Feb 6;132(5):628–44. doi: 10.1161/CIRCRESAHA.122.321956 (PMC9977265; doi:10.1161/CIRCRESAHA.122.321956)
Supplement: Supplementary file 1 [file res-132-628-s001.pdf]

SUPPLEMENTAL MATERIAL

Slower calcium handling balances faster cross-bridge cycling in human *MYBPC3* HCM

Table of contents:

Supplementary materials and methods …………………………………………………page S2

Tables S1 and S2....……………………………………………………………………..page S23

Table S3………………………………………………………………………………page S24

Table S4……………………………………………………………………...….page S25

Table S5………………………………………………………………………………page S26

Figure S1……………………………………………………………………………...page S27

Figure S2……………………………………………………………………………...page S28

Figure S3……………………………………………………………………………...page S29

Figure S4……………………………………………………………………………...page S30

Figure S5……………………………………………………………………………...page S31

Figure S6……………………………………………………………………………...page S32

Figure S7 (uncropped blots)…………………………………………………………..page S33

Examples of statistical analysis………………………………………………………page S34

EXPANDED MATERIALS AND METHODS

CLINICAL STUDY

Patient population

The study conforms with the principles of World Medical Association's Declaration of Helsinki for medical research involving human subjects. The study included 93 patients carrying the *MYBPC3:*c.772G>A (p.Glu258Lys) mutation (see genetic assessment below) identified between January 2001 and December 2019 at the referral center of the Careggi University Hospital, Florence (for clinical details see Table 1). All participants were diagnosed as having HCM by 2-dimensional echocardiographic identification of a hypertrophied (≥15 mm), nondilated LV, in the absence of another cardiac or systemic disease capable of producing that magnitude of ventricular hypertrophy[1]. Echocardiographic studies were performed according to international guidelines[1]. Cardiac magnetic resonance (CMR) imaging, including evaluation of late gadolinium enhancement (LGE), was performed as described[2] using commercially available 1.5-T scanners. The control cohorts for *in vitro* experiments comprised LV specimens from 11 non-transplanted donor hearts, 8 non-failing non-hypertrophic surgical patients (<65 years of age who were undergoing heart surgery for mitral steno-insufficiency, aortic stenosis, or regurgitation). Only patients with absent left ventricular hypertrophy (septal thickness <14 mm) and normal left ventricular systolic function (ejection fraction >55%) were included.

Follow-up and clinical outcomes

Patients were followed up at yearly intervals or more often if clinically indicated, with review of history and symptoms, physical examination, 12-lead electrocardiography (ECG), and

[1] Gersh BJ, Maron BJ, Bonow RO, Dearani JA, Fifer MA, Link MS, et al. 2011 ACCF/AHA guideline for the diagnosis and treatment of hypertrophic cardiomyopathy: executive summary: a report of the American College of Cardiology Foundation/American Heart Association Task Force on Practice Guidelines. Circulation. 2011;124(24):2761-96.
[2] Biagini E, Lorenzini M, Olivotto I, Rocchi G, Lovato L, Lai F, et al. Effects of myocardial fibrosis assessed by MRI on dynamic left ventricular outflow tract obstruction in patients with hypertrophic cardiomyopathy: a retrospective database analysis. BMJ Open. 2012;2(5):e001267.

echocardiographic examination. Additional testing by ambulatory ECG monitoring, exercise testing and cardiac magnetic resonance was performed according to clinical indications. Arrhythmic risk stratification was performed according to international protocols[3].

We systematically collected information regarding clinical outcomes including cardiovascular death, resuscitated cardiac arrest, nonfatal stroke, and progression to severe congestive symptoms (New York Heart Association [NYHA] class III or IV). *Advanced LV dysfunction* was defined by echocardiographic detection of systolic impairment with an ejection fraction <50% (generally used to identify *end-stage* HCM) and/or a restrictive LV filling pattern[4].

Genetic testing and variant classification

After obtaining informed consent, genetic testing was performed. Genetic analysis was executed by Sanger and/or Next Generation Sequencing (NGS). For NGS read alignment, variant calling, and annotation were performed using the MiSeq Reporter and the Variant Interpreter softwares (Illumina, San Diego, CA, USA). All variants reported in this work were sequenced and classified as pathogenic, likely pathogenic, or of uncertain significance following the latest variant interpretation guidelines[5] with the support of Cardio-Classifier. Sequencing for HCM revealed the presence of the *MYBPC3*:c.772G>A (p.E258K) variant in 70 (5.8%) of the 1198 unrelated probands tested at our center until November 2016. In addition, we included in the current analysis 23 other patients carrying the *MYBPC3*:c.772G>A variant comprised of probands tested after November 2016 and/or family members. Allele-specific screening confirmed its absence in 129 healthy controls from the same geographical area. Unfortunately, there are no genome-wide data (WGS / WES / genome-wide SNP

[3] Elliott PM, Anastasakis A, Borger MA, Borggrefe M, Cecchi F, Charron P, et al. 2014 ESC Guidelines on diagnosis and management of hypertrophic cardiomyopathy: the Task Force for the Diagnosis and Management of Hypertrophic Cardiomyopathy of the European Society of Cardiology (ESC). European heart journal. 2014;35(39):2733-79.
[4] Olivotto I, Cecchi F, Poggesi C, Yacoub MH. Patterns of disease progression in hypertrophic cardiomyopathy: an individualized approach to clinical staging. Circulation Heart failure. 2012;5(4):535-46.
[5] Richards S, Aziz N, Bale S, Bick D, Das S, Gastier-Foster J, et al. Standards and guidelines for the interpretation of sequence variants: a joint consensus recommendation of the American College of Medical Genetics and Genomics and the Association for Molecular Pathology. Genetics in medicine: official journal of the American College of Medical Genetics. 2015;17(5):405-24.

array) available for the individuals of the families carrying the mutation, such as the one represented in the pedigree of Figure 1D of the main text. Samples who underwent HCM genetic testing at our center were screened using different targeted gene panels over time (comprising 3,8,12 or 111 genes up until November 2017), and this type of data does not enable linkage studies. On the other hand, we computed a simplified LOD score for the observed co-segregation of the variant with disease using the framework adopted by the ClinGen consortium and described in the publicly available ClinGen Standard Operating Procedures and estimated it as being ~1.2. While this LOD score is not sufficiently high to constitute definitive evidence of pathogenicity for the variant in question, c.772G>A is a known HCM-causing variant and it is classified as such according to the the American College of Medical Genetics and Genomics (ACMG) variant interpretation framework; the variant is classified as pathogenic by the CardioClassifier tool, activating guideline criteria PP1, PP2, PM2, PS3, PS4. Hence, this variant is considered causal in all instances in which it's detected in an HCM patient, irrespective of segregation in his/her family.

Microsatellite and haplotype analysis on carriers of the pathogenic MYBPC*3:P.Glu258Lys* variant

Haplotype analysis was performed using the Merlin software[6] on 4 selected informative multi-generational pedigrees in which the variant segregated with HCM, analysing 4 SNPs (rs3729989, rs11570058, rs3729953 and rs1052373) and 5 STRs (MYBPC3_CA_2, MYBPC3_CA_3, MYBPC3_CA_4, MYBPC3_GT_1 and D11S1344). Details regarding primers used for amplification and genotyping techniques are available in Table S1. Segregation analysis was subsequently used to infer the individual haplotypes (Fig. 1).

***IN VITRO* STUDIES**

Patients for *in vitro* studies

---

[6] Abecasis GR, Cherny SS, Cookson WO, Cardon LR. Merlin—rapid analysis of dense genetic maps using sparse gene flow trees. Nature Genetics. 2002;30(1):97-101.

In vitro studies were performed at the University of Florence. Protocols were approved by the ethical committee of Careggi University-Hospital (2006/0024713; renewed May 2009). *In vitro* studies were performed on cardiac samples from four c.772G>A patients consecutively referred to surgical myectomy for relief of drug-refractory symptoms related to LV outflow tract obstruction. Clinical details of these four patients (ID1, ID2, ID3, ID4) are given in Table S1.

Tissue processing

Septal specimens were prepared as we previously described[7]. Briefly, septal samples from HCM and control patients were rapidly washed in ice-cold cardioplegic solution containing (in mmol/L): KH_2PO_4 50, $MgSO_4$ 8, HEPES 10, adenosine 5, glucose 140, mannitol 100, taurine 10 (pH 7.4 with KOH). Within 15 minutes from excision, a small portion of the tissue was frozen in liquid nitrogen and used for protein isolation. The remaining fresh tissue was kept in ice-cold cardioplegic solution and used to isolate multicellular preparations, single myofibrils and single cardiomyocytes. Endocardial trabeculae suitable for mechanical measurements (300-800 µm diameter) were dissected, while the remaining tissue was minced to small pieces (~1mm^3) and subjected to enzymatic and mechanical dissociation to obtain viable single myocytes, as described before[7]. In brief, tissue chunks were dissected into small pieces (~1mm^3) in a scraping device and the bathing solution changed to Ca^{2+}-free dissociation buffer containing (in mM): NaCl 113, KCl 4.7, KH_2PO_4 0.6, Na_2HPO_4 0.6, $MgSO_4$-$7H_2O$ 1.2, $NaHCO_3$ 12, $KHCO_3$ 10, HEPES 10, taurine 20, Na pyruvate 4, glucose 10, BDM 10 (pH 7.3 with NaOH) and heated to 37 ºC. Collagenase Type V and Protease Type XXIV (Sigma) were subsequently added, and tissue chunks digested for a total of 2 hours. During the digestion, the buffer containing dissociated myocytes was collected every 15 minutes from the scraping device and diluted with KB solution at room temperature (RT). KB solution contained (in mM): KCl 20, KH_2PO_4 10, glucose 25, mannitol 5, L-glutamic acid monopotassium salt 70, β-hydroxybutyric acid 10, EGTA

[7] Coppini R, Ferrantini C, Yao L, Fan P, Del Lungo M, Stillitano F, et al. Late Sodium Current Inhibition Reverses Electromechanical Dysfunction in Human Hypertrophic Cardiomyopathy. Circulation. 2013;127(5):575-84.

10 and 2mg/mL albumin (pH 7.2 with KOH). The myocytes were left to settle and then resuspended in Ca^{2+}-free Tyrode solution containing (in mM): 132 NaCl, 4 KCl, 1.2 MgCl2 10 HEPES, and 11 glucose (pH 7.35 NaOH). $CaCl_2$ was added stepwise up to 0.6 mM. Cells were stored in this solution and used within 3 hours.

Simultaneous energetic and mechanical measurements in skinned ventricular multicellular strips

Ventricular muscle strips were dissected parallel to the long axis of the fibres in cold relaxing solution under a dissecting microscope. Subsequently, muscle strips were chemically permeabilised by overnight incubation in relaxing solution with 1% Triton X-100 at 4 °C. Tissues were then extensively washed in fresh ice-cold relaxing solution, stored at 4 °C, and used within 24 hours. Experimental solutions and equipment's used were as described previously[8]. Briefly, the skinned preparations were mounted horizontally between a force transducer and a length controller via aluminium T-clips. The length of the preparations was adjusted based on the passive tension stretching them about 15% below the slack length, which correspond to a sarcomere length of about 2.1-2.2 µm. Mean dimensions (± S.E.M.) of HCM and donor ventricular preparations amounted to 1.34±0.06 and 1.53±0.07 mm in length, 0.31±0.01 and 0.27±0.01 µm in width, and 0.28±0.01 and 0.23±0.01 µm in depth, respectively. Isometric force and ATPase activity were simultaneously measured at saturating (pCa 4.5) and sub-saturating Ca^{2+} concentrations at 25 °C. The skinned fibers were activated inside a small (30 µl) chamber with quartz windows containing activating solution, and force generated and ATP consumed were measured simultaneously during each contraction[9].

[8] Coppini R, Ferrantini C, Aiazzi A, Mazzoni L, Sartiani L, Mugelli A, et al. Isolation and functional characterization of human ventricular cardiomyocytes from fresh surgical samples. J Vis Exp. 2014(86):51116.
[9] Witjas-Paalberends ER, Ferrara C, Scellini B, Piroddi N, Montag J, Tesi C, et al. Faster cross-bridge detachment and increased tension cost in human hypertrophic cardiomyopathy with the R403Q MYH7 mutation. The Journal of physiology. 2014;592(15):3257-72.

Maximal force was determined at steady-state level and normalized to the cross-sectional area (CSA, mm^2) of the muscle strip to calculate tension (force/CSA, mN/mm^2). The CSA of the preparation was estimated based on an elliptical shape, i.e. CSA = (width x depth x π) / 4. The muscle was manually transferred between different baths and exposed to a range of activating calcium so that the pCa-force relationship could be determined. Tension values at different pCa were fitted with a modified Hill equation and the concentration of Ca^{2+} required to reach the half of maximum force (pCa_{50}) was estimated to determine myofibril-Ca^{2+} sensitivity[10]. Solutions with intermediate [Ca^{2+}] were obtained by appropriate mixing of the activating and relaxing solutions.

ATPase activity was measured by an UV-coupled optical absorbance enzyme assay[11]. Briefly, ATP hydrolysis, inside the muscle, was coupled to the oxidation of NADH to NAD^+ catalyzed by pyruvate kinase and L-lactic dehydrogenase. NADH oxidation, and thus ATP consumption, was measured photometrically from the absorbance at 340 nm of near-UV light. This absorbance signal was calibrated using multiple injections of 50 nl of 10 mM ADP in the measuring bath. Using this calibration, ATPase activity ($pmol*ul^{-1}*s^{-1}$) in the preparation was derived from the slope of the absorbance signal. Maximal Ca^{2+} activated ATPase activity at each [Ca^{2+}] was calculated by subtracting basal rate of ATPase activity in relaxing solution (pCa10) and normalizing to the volume (length x CSA, mm^3) of the muscle strip. Tension cost (isometric ATPase per unit force, $pmol*ul^{-1}*s^{-1}/mN/mm^2$) was calculated from the slope of the linear relationship between mean values of tension and ATPase activity once all the data were pooled in 10% wide normalized steady state force bins or as the ratio between maximal isometric tension and maximal ATPase activity.

Strip *ex vivo* preparation: fixation, staining and clearing

[10] Wijnker PJ, Sequeira V, Witjas-Paalberends ER, Foster DB, dos Remedios CG, Murphy AM, et al. Phosphorylation of protein kinase C sites Ser42/44 decreases Ca(2+)-sensitivity and blunts enhanced length-dependent activation in response to protein kinase A in human cardiomyocytes. Archives of biochemistry and biophysics. 2014;554:11-21.

[11] Narolska NA, van Loon RB, Boontje NM, Zaremba R, Penas SE, Russell J, et al. Myocardial contraction is 5-fold more economical in ventricular than in atrial human tissue. Cardiovascular research. 2005;65(1):221-9.

The comparison of mechanical and energetic measurements between donor and mutant trabeculae is meaningful if cardiomyocyte orientation along the muscle strips and mechanical conditions of contraction are the same for both groups of preparations. Altered cardiomyocyte alignment and myofibril "disarray", described patchily in HCM myocardium[12], may reduce isometric tension while increasing the ATP consumption, due to larger 'internal shortening'.

To perform structural analysis of the experimental preparations, the cardiac strips were fixed overnight in 5 ml of 4% paraformaldehyde (PFA) at 4°C. During fixation, samples were kept stretched to maintain the same structural organization of the tissue and avoid swelling. Samples were then rinsed three times (15 minutes each) in 5 ml of 0.01M phosphate buffered saline (PBS) at 4°C (pH 7.6). Each strip was stained first with an anti-α-actinin antibody (Ig_1) (A7811, Sigma-Aldrich, US, dilution 1:200) for 1 day at 4°C, then, after one day of washing with PBST 0.1% at room temperature, a secondary antibody (Ig_2) conjugated with an Alexa Fluor 594 (ab150108, Abcam, UK, dilution 1:100) was applied for 3 days at room temperature. The staining protocol used on human samples had been previously optimized on three-month-old C57Bl/6 wild-type (WT) mouse hearts[13]. The staining protocol with the highest signal amplification for the human permeabilized strips corresponded to incubation times of 24 and 72 hours for Ig_1 and Ig_2, respectively.

Then, after one day of washing with PBST 0.1% at room temperature, samples were fixed in 1 ml of PFA 4% for 5 minutes to prevent antibody detachment. To remove PFA residue, samples were rinsed three times (5 minutes each) in 1 ml of 0.01M PBS (pH 7.6, room temperature). Finally, samples were optically cleared by homogenizing the refractive index of the tissue using serial incubations in 2 ml of 20%, 47% and 68% (vol/vol) 2,2'-thiodiethanol (166782-500G, Sigma-Aldrich, US) in 0.01 M PBS (TDE/PBS) each for 1 hour at room temperature while gently shaking (protocol modified from that described in[13]).

---

[12] Poggesi C, Ho CY. Muscle dysfunction in hypertrophic cardiomyopathy: what is needed to move to translation? Journal of muscle research and cell motility. 2014;35(1):37-45.

[13] Costantini I, Ghobril JP, Di Giovanna AP, Allegra Mascaro AL, Silvestri L, Mullenbroich MC, et al. A versatile clearing agent for multi-modal brain imaging. Scientific reports. 2015;5:9808.

Imaging and image pre-processing

A custom-made two-photon fluorescence microscope (TPFM) was used to image the strips. A mode locked Ti:Sapphire laser (Chameleon, 120 fs pulse width, 90 MHz repetition rate, Coherent, CA) operating at 780 nm was coupled into a custom-made scanning system based on a pair of galvanometric mirrors (LSKGG4/M, Thorlabs, USA). The laser was focused onto the specimen by a refractive index tunable 25X objective lens (LD LCI Plan-Apochromat 25X/0.8 Imm Corr DIC M27, Zeiss, Germany). The system was equipped with a closed-loop XY stage (U-780 PILine® XY Stage System, Physik Instrumente, Germany) for radial displacement of the sample and with a closed-loop piezoelectric stage (ND72Z2LAQ PIFOC objective scanning system, 2 mm travel range, Physik Instrumente, Germany) for the displacement of the objective along the z axis. The fluorescence signal was collected by an independent GaAsP photomultiplier module (H7422, Hamamatsu Photonics, NJ). Emission filter of 618 ± 25 nm was used for Alexa Fluor 594 detection.

The whole volume was acquired performing z-stack imaging of adjacent regions. Each stack had the depth equal to the thickness of the analyzed strip (in a range of 250-350 μm) with a z step of 2 μm between images. Each frame had a field of view of (450 × 450) μm and a pixel size of (0.44 × 0.44) μm. Adjacent stacks had an overlap of 40 μm. Stitching was therefore performed using a software tool for high-resolution volumetric stitching (https://github.com/lens-biophotonics/ZetaStitcher). This software exploits the overlap between neighboring stacks to correct the micron-scale mechanical error of the imaging platform. It is based on two steps: an alignment process followed by image fusion. As a first step, a 2D cross-correlation map was evaluated at several depths for every pair of adjacent 3D stacks, moving each stack relative to its neighbor. The final position of all stacks was determined by applying a global optimization algorithm to the displacements of the individual pairs. Finally, the stacks were fused into a 3D reconstruction of the whole sample stored in single TIFF file. To increase the resolution of the sarcomere structures, a deconvolution routine was performed using the Huygens Software (Scientific Volume Imaging) based on the actual Point Spread Function (PSF)

of our TPFM (Fig. 3B). The deconvolved data is obtained using the Deconvolution Wizard with default settings. Fig. 3B shows a zoom in of original and deconvolved image.

Then, a Contrast Limited Adaptive Histogram Equalization (clip = 0.08, K = 128 pixels) was applied to each frame. Finally, to perform a 3D segmentation of the muscle tissue, a 2D analysis was applied at each optical section where a K-Means algorithm was used to quantize the pixel intensities in four levels. These four clusters were dynamically classified as background or tissue and morphological operators were applied to the binary segmentation in order to delete artifacts and to smooth the result. Specifically, we applied the closing operator to remove the salt and pepper noise artifacts; then, we filled little black holes and removed little white objects to include intracellular space and to remove extracellular elements; finally, we applied an opening operator and a dilatation operator to smooth the border of the cell areas. The 3D mask was then obtained by stacking 2D segmented sections along the Z axis and applied the TPFM stitched image.

Mechanical Measurements in Myofibrils

Single myofibrils or small bundles of myofibrils were isolated from LV human samples and used for mechanical experiments as previously described[14]. Thin strips of the ventricular samples were incubated overnight in cold relaxing solution (pCa9) with 0.5% Triton X-100[15]. After Triton removal, the strips were homogenized in relaxing solution to obtain a myofibril suspension that was stored at 4°C. Myofibrils were mounted in a custom-built apparatus (pCa9, 15 °C) and mounted between two glass micro-needle, one connected to a high-speed length controller and the other one acting as a calibrated cantilever force probe. The initial sarcomere length was set at 2.2 µm. Activating and

---

[14] Piroddi N, Belus A, Scellini B, Tesi C, Giunti G, Cerbai E, et al. Tension generation and relaxation in single myofibrils from human atrial and ventricular myocardium. Pflugers Archiv : European journal of physiology. 2007;454(1):63-73.

[15] Belus A, Piroddi N, Scellini B, Tesi C, D'Amati G, Girolami F, et al. The familial hypertrophic cardiomyopathy-associated myosin mutation R403Q accelerates tension generation and relaxation of human cardiac myofibrils. The Journal of physiology. 2008;586(15):3639-44.

relaxing solutions calculated as previously described[14,15], were at pH 7.0 and at ionic strength 200 mmol/L. Myofibrils were maximally activated and fully relaxed by rapid switching (<5 ms) between two continuous streams of activating (pCa4.5) and relaxing (pCa9) solutions flowing from a double-barreled glass pipette. Fast solution switching technique allowed for the measurements of steady-state isometric force as well as the rate of force activation and relaxation. Maximal isometric force (mN) was normalized to the cross-sectional area of the myofibril (mm^2). The time required to reach 50% of the maximal force was used to estimate the rate constant of force development, k_{ACT} (s^{-1}), and the rate constant of force redevelopment following release/restretch protocol applied at steady state force, k_{TR} (s^{-1}). The kinetics of the two phases of relaxation were estimated as follows: 1) the rate constant of the slow early phase of relaxation, slow k_{REL} (s^{-1}), was calculated from the slope of the regression line fitted to the tension trace normalized to the entire tension relaxation transient; 2) the rate constant of the final fast phase of relaxation, fast k_{REL} (s^{-1}), was measured from a mono-exponential fit.

All solutions contained a cocktail of protease inhibitors: 10 mM leupeptin, 5 mM pepstatin A, 200 mM phenyl-methylsulphonylfluoride, 10 mM E64, 500 mM NaN3 and 2 mM dithiothreitol. The level of contaminant inorganic phosphate in the solutions was kept less than 5 umol/L using a phosphate scavenging system (purine-nucleoside-phosphorylase with substrate 7-methyl-guanosine)[16]. The regeneration of MgATP inside the solutions was achieved adding Creatine phosphate (10mM) and creatine kinase (200 unit ml^{-1}).

Current-clamp/intracellular Ca^{2+} studies on single myocytes

These experiments were conducted as we previously described[16]. Myocytes were incubated for 30 minutes with the Ca^{2+} indicator Fluoforte (Enzo Life Sciences, Farmingdale, New York) at RT, washed, and transferred to the microscope-mounted recording chamber. Fluoforte fluorescence was detected at 505-520nm (using a high-speed high-sensitivity EMCCD Camera, model Evolve Delta

---

[16] Tesi C, Piroddi N, Colomo F, Poggesi C. Relaxation kinetics following sudden Ca(2+) reduction in single myofibrils from skeletal muscle. Biophys J. 2002;83(4):2142-51.

by Photometrics, USA), during bright-field illumination at 492±3 nm (using a dedicated LED light source, model SpectraX by Lumencor). Experimental temperature was 35±5 °C for all protocols. Intracellular Ca^{2+} was monitored during field stimulation at different pacing rates and under acute administration of drugs as detailed below. Action potentials (APs) were measured simultaneously using the perforated-patch configuration (amphotericin-B method). Specifically, for AP recordings, the pipette solution contained (in mM) 115 K methanesulfonate, 25 KCl, 10 HEPES, 3$MgCl_2$ and cells were superfused with Tyrode buffer (see above) containing 1.8mM $CaCl_2$. APs were elicited with short depolarizing stimuli (<3ms) at different frequency of stimulation (0.2Hz, 0.5Hz and 1Hz, 1 minute at each frequency).

Intact trabeculae studies

Intact ventricular trabeculae were mounted between a basket-shaped end of a force transducer (KG7A, Scientific Instruments Heidelberg, Germany) and a motor (Aurora Scientific Inc., Aurora, Canada), controlled by a custom Labview (National Instruments, Austin, Texas) program. Muscles were mounted in cold cardioplegic solution and then perfused with Krebs/Henseleit buffer, containing (in mM) 119 NaCl, 4.7 KCl, 2.5 $CaCl_2$, 1.2 $MgSO_4$, 1.2 KH_2PO_4, 25 $NaHCO_3$; pH 7.4 with 95%O2:5%CO2. Muscles were allowed to stabilize for at least 30 min before recordings. Diastolic sarcomere length was assessed by calculating the average distance of striations and set at 2.10-2.20 µm. Isometric force was recorded at 35±2°C under various conditions. In brief, inotropic responses to increased pacing frequencies, stimulation pauses, and beta-adrenoceptor agonist isoproterenol (10-7M) were evaluated, and kinetics of isometric twitches was assessed under all conditions. The trabecula was stimulated at increasing pacing rates (0.1-2.5 Hz): at each frequency, force was allowed to reach steady-state before recordings. Stimulation pauses (30s) were inserted after the last contraction of a steady series (at 0.5 Hz) and post-rest potentiation was evaluated at the first stimulated beat after the pause. Finally, muscle section was measured for force normalization.

RT-PCR:

RT-PCR: Total mRNA was isolated from frozen human septal specimens using the RNeasy Fibrous Tissue Mini Kit (Qiagen) following manufacturer's instructions. Subsequently, complementary DNA (cDNA) was synthesized from 1 μg of total RNA using iScript ™ cDNA Synthesis kit (Bio-Rad Laboratories S.r.l.). All these steps were performed according to the manufacturer's instructions.

Real-time quantitative PCR (Q-PCR) was performed using iTaq™ SYBR® Green Universal Supermix and predesigned primers assays for the following genes: KCNQ1, KCNH2, KCND3, KCNIP2, KCNE1 (Bio-Rad Laboratories S.r.l.).

All reactions were performed in triplicate and included a negative control. Relative quantification of the mRNA level for the different genes was determined by the Bio-Rad CFX Maestro software (Bio-Rad Laboratories S.r.l.), using the comparative method (ΔΔCt). In brief, the threshold cycle (Ct) difference of the index gene and the reference gene, calculated from each specimen, is subtracted from the average Ct of the control group; this value is used as the exponent to calculate ΔΔCt for each specimen. For all mRNA quantification assay, the housekeeping gene 18S ribosomal RNA was used as the reference gene.

Western Immunoblotting on human myectomy samples

Frozen cardiac samples were treated with trichloroacetic acid before protein analysis. To quantify total cMyBP-C protein levels and confirm that truncated proteins are not detectable, total protein homogenates were made from frozen cardiac tissue as described before[17]. Ten µg of protein lysate was separated by one-dimension gel electrophoresis on a 4-15% gradient polyacrylamide gel and subsequently transferred to a polyvinylidene difluoride (PVDF) membrane by semi-dry blotting. After blocking non-specific binding with 5% non-fat milk powder in Tris-buffered saline with 0.1% (v/v) Tween-20 (TBS-T), cMyBP-C protein levels were detected by incubating with a polyclonal antibody (diluted 1:1000) raised against the amino acid 2-14 of cMyBP-C (a gift from Dr. Sakthivel Sadayappan, UC College of Medicine, Cincinnati). This antibody should be able to detect both

healthy and mutant protein. Secondary goat anti-rabbit horseradish peroxidase (HRP) conjugated antibody (1:1000) binding was visualized by enhanced chemiluminescence (Amersham, GE Healthcare, Chalfont St. Giles, UK). α-actin was used as a loading control and donor cMyBP-C/α-actin levels (control group) were set to 1.

SYPRO Ruby and ProQ Diamond Staining of Gradient Gels

Proteins were separated on 4% to 15% precast Tris-HCl gels (Bio-Rad Laboratories, Hercules, Calif) and stained with SYPRO Ruby and ProQ Diamond to determine sarcomeric protein levels and phosphorylation, respectively, as described previously[17]. The phosphorylation status of sarcomeric proteins was expressed relative to α-actinin, except when noted otherwise. Protein values of c.772G>A samples are given as fraction (or percentage) of the value found for donor samples, which was set to 1 (or 100%).

Patient stem cell line generation and quality control

Patient blood samples were collected at the University of Florence with informed consent (protocol SILICO-FCM 777204 approved on the 04-15-2019 by the ethical committee of Regione Toscana). Peripheral blood mononuclear cells (PBMC) were isolated using a standard isolation method. PBMCs were isolated by density gradient centrifugation Ficoll (sodium diatrizoate, polysaccharides, and water, density of 1.08 g/mL) in equal volume of whole blood diluted in PBS and centrifuged for 40 minutes at 400-500 g without brake. The “ring” of PBMCs were collected at $2x10^6$ cell/ml in 20% dimethyl sulfoxide (DMSO) and 90% fetal bovine serum (FBS) and placed inside a freezing container (Nalgene Mr. Frosty) at -80°C overnight to allow gradual cooling and moved to liquid nitrogen the following day. PBMCs were shipped to the University of Washington where they were reprogrammed into human induced pluripotent stem cells (hiPSCs) utilizing the CytoTune®-iPS 2.0 Sendai

[17] van Dijk SJ, Dooijes D, dos Remedios C, Michels M, Lamers JM, Winegrad S, et al. Cardiac myosin-binding protein C mutations and hypertrophic cardiomyopathy: haploinsufficiency, deranged phosphorylation, and cardiomyocyte dysfunction. Circulation. 2009;119(11):1473-83.

Reprogramming Kit (Gibco). Two representative clones per patient iPSC line were selected based on flow cytometry of four pluripotency markers (Oct4, Nanog, SSEA-4, and TRA-1-60) (Fig.S4-A). Further quality control of these patient clones was performed including karyotyping (Cytogenetics) (Fig.S4-B), Sanger sequencing for the for the single nucleotide substitution at position 772 (GeneWiz) (Fig.S4-C), and STEMdiff trilineage differentiation/qPCR array (STEMCELL Technologies) to confirm ability to differentiation into all three germ layers (data not shown). A representative patient line was genome edited using CRISPR/Cas9 (Clustered Regularly Interspaced Short Palindromic Repeats/Cas9) to create a homozygous isogenic control line. hiPSC lines utilized for this work include, WTC11 line engineered by Dr. Bruce Conklin (The Gladstone Institute), the ID3 patient iPSC line and the corrected ID3 isogenic control line.

Human induced pluripotent stem cell derived cardiomyocyte culture and differentiation

All three undifferentiated hiPSC lines (WTC11, UC3-4, ID3-clone 1 and corrected-ID3-clone 3) were maintained on 1X Matrigel lot -3013 (Corning, 5.1mg/ml concentration at 30X) with mTeSR1 media (STEMCELL Technologies). At the University of Washington laboratory, a well-established small molecule directed differentiation protocol was used with CHIR99021 (Cayman Chemicals) induction on day 0 to differentiate hiPSCs to human induced pluripotent stem cell derived cardiomyocytes (hiPSC-CMs)[18]. On day 14, hiPSC-CMs were replated for cardiomyocyte enrichment utilizing lactate purification in glucose starved media for 4 days (4mM sodium lactate, Sigma, in RPMI media without glucose, Thermo Fisher), after which cells were returned to cardiomyocyte maintenance media. Similarly, in the Florence laboratory undifferentiated hiPSC cell lines were expanded under serum-free conditions in mTeSR medium (Stem Cell Technolgies, Vancouver, Canada) on a Matrigel matrix (Matrigel® hESC-Qualified Matrix, Corning®, New York, NY, USA), at 37°C, 5% CO_2 and for

[18] Lian X, Zhang J, Azarin SM, Zhu K, Hazeltine LB, Bao X, et al. Directed cardiomyocyte differentiation from human pluripotent stem cells by modulating Wnt/beta-catenin signaling under fully defined conditions. Nature protocols. 2013;8(1):162-75.

cardiac differentiation a monolayer directed differentiation protocol on Matrigel matrix (Matrigel® hESC-Qualified Matrix, Corning®, New York, NY, USA) coated 24-well plate, using the cardiac PSC Cardiomyocyte Differentiation Kit (Life Technologies, Thermo Fisher scientific, Carlsbad, CA, USA). Reached a confluence of 70-80%, hiPSC colonies were chemically dissociated using 1x Tryple (Life Technologies, Thermo Fisher scientific, Carlsbad, CA, USA), suspended into mTeSR with 5µM of Y-27632 ROCK inhibitor (Stem Cell Technologies, Vancouver, Canada). The single cells were seeded in each well of a 24-well plate at cell density of 60,000-80,000 cell/well (depending on the cell line). At 70% of confluency (~2 days after dissociation) the medium was changed to Cardiomyocyte Differentiation Medium A (referred to as day 0 of the differentiation protocol). Two days later, Medium A was replaced with Medium B and after a further 2 days with Medium C, which was subsequently changed every 2 days, until spontaneously beating appear (day 8 of differentiation). On day 12, Medium C was replaced with RPMI plus B-27 supplement (Life Technologies, Thermo Fisher scientific, Carlsbad, CA, USA). On day 15 post differentiation, single hiPSC cardiomyocytes were isolated by enzymatic dissociation with Tryple (Life Technologies, Thermo Fisher scientific, Carlsbad, CA, USA). The hiPSC-CMs obtained were either seeded on hydrogel-based micropatterned surfaces, to improve cardiac maturation, or used for EHTs generation. The two distinct protocols of our laboratories did not differ the quality of cardiac differentiation.

Whole cell protein analysis

hiPSC-CMs cultured in a monolayer were collected at day 14-, 30-, 45-, and 60-days post initiation of differentiation and spun down at 1000rpm for 5 mins. Supernatant was removed and cell pellets were immediately frozen in liquid nitrogen. Cell pellets were then stored at -80°C. Protein was isolated from these cell pellets utilizing lab made lysis buffer (150mM NaCl, 0.1% Triton X-100, 50mM Tris-HCl at pH 8.0) with 1:100 protease inhibitor cocktail (Sigma). Protein concentration was quantified utilizing the Bradford Assay (BioRad, 5x stock) and BSA standards (2mg/ml stock, Thermo Fisher) on a plate reader measuring absorbance at 595nm. Western blot (WB) was performed

to probe for proteins of interest. Briefly, 20µg of protein per sample was diluted in 4x Laemmli buffer (BioRad) and denatured at 95°C for 5 mins. Samples were separated using 4-20% gradient Mini-PROTEAN® TGX Stain-Free Protein Gels (BioRad) and then transferred onto Immuno-Blot® LF PVDF Membranes (BioRad). Membranes were blocked in Blocking Buffer for Fluorescent Western Blotting (Rockland). Primary antibodies incubated overnight: cMyBP-C (1:2000, gift from Dr. Samantha Harris, University of Arizona, Tucson, AZ, USA), α-actinin (1:300, Abcam, ab9465), and GAPDH (1:2000, Invitrogen, AM4300). Secondary antibodies, Alexa Fluor 488 and 647, were diluted 1:2000 in blocking buffer for 1h at RT (Life Technologies). Antibody stripping was performed to quantify multiple proteins from the same blot. Restore™ western blot stripping buffer (Thermo Scientific) was added to membranes and incubated at RT for 30 mins and then washed off with TBS-T (3x). Membranes were re-blocked prior to subsequent primary antibody incubation. Fluorescent western blots were imaged utilizing a ChemiDoc MP (BioRad) and quantified using FIJI (Fig.S5).

HiPSC-CMs maturation on biomimetic hydrogels

On day 15-20 of differentiation, single hiPSC-CMs previously exposed to 1h of ROCK inhibitor 10µM were collected with Tryple-dissociation, resuspended in RPMI/B27 and 10µM Y-27632 ROCK inhibitor (Stem Cell Technologies, Vancouver, Canada), and seeded onto diethylene glycol diacrylate (DEG-DA) micropatterned substrates at the density of 20,000 cells/cm^2, as previously described[19]. Dual recording experiments of long-term cultured hiPSC-CMs were performed at day 60 post-differentiation.

Freezing and thawing of hiPSC-cardiomyocytes

[19] Pioner JM, Santini L, Palandri C, Martella D, Lupi F, Langione M, et al. Optical Investigation of Action Potential and Calcium Handling Maturation of hiPSC-Cardiomyocytes on Biomimetic Substrates. International journal of molecular sciences. 2019;20(15).

For hiPSC-CM freezing, single hiPSC-CMs previously exposed to 1h of ROCK inhibitor 10μM were collected with Tryple-dissociation and resuspended in KO-DMEM (1:1), centrifuged at 1000 rpm for 5 minutes. Cells were rapidly resuspended in CryoStor© CS10 freezing medium and frozen in cryovials at 1°C/min at -80°C for at least overnight before moving to liquid nitrogen.

Cell fractional shortening

Single hiPSC-CM fractional shortening was visualized using a Nikon TS100 inverted microscope coupled to a video-based edge detection system at 40x of magnification (Olympus). For measurements of single cell fractional shortening, day 20 post differentiated single hiPSC-CMs were re-plated onto 10μg/ml human fibronectin-coated surfaces (35mm diameter, Fisher) and measured at day 60 post differentiation. All cells were perfused at 37°C with a Tyrode solution (in mM: NaCl 138, KCl 3.7, HEPES 20, KH_2PO_4 1.2, $MgSO_4 \cdot 7H_2O$ 1.2, Glucose 5, $CaCl_2 \cdot 2H_2O$ 1.8, pH 7.0) subjected to a sequential pacing train of at least 10 seconds at 1Hz. Traces were analyzed using the IonWizard (IonOptix) software. A minimum of five traces were analyzed and averaged for each cell. Spontaneously beating hiPSC-CMs were excluded from this analysis.

Generation and mechanical measurements of Human Engineered Heart Tissues

For the generation of EHTs, cells were used when hiPSC-CMs were at ~90% confluency. On day 15 of differentiation culture, beating cells were washed with 1x PBS and dissociated with 1x Tryple (Life Technologies, Thermo Fisher scientific, Carlsbad, CA, USA) for 10 min at 37°C. Gently, the cell suspension was transferred into a centrifuge tube with RPMI/B27 medium supplemented with 10% Fetal Bovine Serum (FBS)(Life Technologies, Thermo Fisher scientific, Carlsbad, CA, USA). HiPSC-CMs were centrifuged at 1000 RPM for 5 min and resuspended in cardiac medium with 10%

FBS. Human EHTs were generated following previously described protocols[20 21]. Briefly, agarose casting molds were prepared in 24-well tissue culture plates with 2% Agarose (Life Technologies, Thermo Fisher scientific, Carlsbad, CA, USA), dissolved in the appropriate volume of PBS, and custom-made Teflon spacers (EHT Technologies GmbH, Hamburg, Germany). After agarose solidification (~10 min), the Teflon spacers were removed and silicone racks (EHT Technologies GmbH, Hamburg, Germany) were positioned upside down in the middle of each agarose wells. EHTs were generated with 100µl per EHT, consisting of 1 x 10^6 cells in RPMI/B27 with 5 mg/ml Fibrinogen from bovine plasma (Sigma-Aldrich) and 3 U/mL Thrombin (Sigma-Aldrich). The mixture was pipetted into the agarose casting molds and incubated for 80 min at 37°C, 5% CO_2. After fibrin polymerization, the EHTs were transferred into fresh culture medium consisting of RPMI plus B27 supplement, 10% FBS and 33µg/ml Aprotinin (Sigma-Aldrich). EHT medium was changed every 2 days until experimental days, and contractile analysis was performed on 50-day-old EHTs. Thawed hiPSC-CMs were used to generate EHTs and no differences were found. Auxotonic spontaneous contraction and frequency were regularly measured over a period of 50 days in culture (Fig. S3) by recording the deflection of individual post using a 10x of magnification (Olympus) using an Evos FL2 auto system (20 seconds, 32 frame/sec)(Life Technologies) with an on-stage incubator (37°C, 5% CO_2, in RPMI/B27 ~0.4Mm of Ca^{2+}) connected to a BenchPro 2100 Plasmid Purification System (Invitrogen). The relationship between the deflection of the individual pillars and force is 0.28 µN/µm. At day 50, EHTs are manually detached from the silicon pillars, mounted, and measured for the mechanical properties in the same conditions described for patient intact cardiac muscle. EHTs were adapted and measured at 0.5, 0.8, 1, 1.2, 1.8, 2, 3, and 4mM of Ca^{2+} concentration.

[20] Mannhardt I, Breckwoldt K, Letuffe-Breniere D, Schaaf S, Schulz H, Neuber C, et al. Human Engineered Heart Tissue: Analysis of Contractile Force. Stem cell reports. 2016;7(1):29-42.

[21] Mannhardt I, Saleem U, Benzin A, Schulze T, Klampe B, Eschenhagen T, et al. Automated Contraction Analysis of Human Engineered Heart Tissue for Cardiac Drug Safety Screening. Journal of visualized experiments : JoVE. 2017(122).

IN SILICO analysis

In silico human-based electromechanical simulations

Mechanistic simulations of human ventricular myocyte electromechanical function were conducted to explain the impact of altered crossbridge cycling on force generation, in the context of HCM ionic remodeling.

The endocardial cell version of the electromechanical model published in [22] was adopted, which describes human ventricular myocyte excitation-contraction coupling [23] and force generation [24]. Simulations were conducted in MatLab (Mathworks Inc. Natick, MA, USA) using the ordinary differential equation solver ode15s. A stimulus current of -53 µA/µF with 1 ms duration was applied and steady-state was reached at 1 Hz pacing. First, the effect of altered crossbridge cycling on force generation was analyzed. For this, a scaling factor of 2, consistent with experimental evidence, was applied to the model transition rates k_{ws} and k_{su}. In the Land model of human cardiac contraction, these drive the transitions from the pre-powerstroke to the post-powerstroke state and from the post-powerstroke to the detached state [24], respectively. Then, the effect of altered crossbridge cycling was evaluated in the context of HCM ionic remodeling. This was implemented based on [25], as illustrated in Table S5. Simulated action potential, calcium transient, and active tension waveforms were compared to experimental data. Of note, since we miss specific information of the mechanisms that underly AP/calcium transient prolongation with this specific mutation, we mimicked the c.772G>A excitation-contraction coupling remodeling according to the ionic remodeling described in Coppini et al., 2013 for cardiomyocytes from HCM with different genetic backgrounds.

---

[22] Margara F, Wang ZJ, Levrero-Florencio F, Santiago A, Vázquez M, Bueno-Orovio A, et al. In-silico human electro-mechanical ventricular modelling and simulation for drug-induced pro-arrhythmia and inotropic risk assessment. Progress in Biophysics and Molecular Biology. 2021;159:58-74.

[23] Tomek J, Bueno-Orovio A, Passini E, Zhou X, Minchole A, Britton O, et al. Development, calibration, and validation of a novel human ventricular myocyte model in health, disease, and drug block. eLife. 2019;8.

[24] Land S, Park-Holohan SJ, Smith NP, Dos Remedios CG, Kentish JC, Niederer SA. A model of cardiac contraction based on novel measurements of tension development in human cardiomyocytes. Journal of molecular and cellular cardiology. 2017;106:68-83.

[25] Passini E, Mincholé A, Coppini R, Cerbai E, Rodriguez B, Severi S, et al. Mechanisms of pro-arrhythmic abnormalities in ventricular repolarisation and anti-arrhythmic therapies in human hypertrophic cardiomyopathy. Journal of molecular and cellular cardiology. 2016;96:72-81.

Statistical Analysis

All group data are expressed as mean ± Standard Error of Mean (SEM and the number of patients (*N*) and the number of samples (*n*; i.e., the number of LV strips or myofibrils that underwent a specific measurement) are indicated in the respective legends.

Data from studies on isolated myocytes, trabeculae and proteins are expressed and plotted as means ± SEM values obtained from a number of independent determinations on different myocytes or muscles: number of cells/trabeculae (*n*) and number of patients (N) are indicated in the figure legends for each set of measurements.

To faithfully compare different sets of measurements, sensitivity analysis was performed for each statistical comparison, in order to account for:

1- non Gaussian distribution

The data were tested for normality using the Skewness/Kurtosis test[26].

2- heteroschedasticity (inequality of variances)

We used the F test for equality of variances in two-group comparison studies and the Bartlett's test for variance homogeneity in the multiple comparison design.

Non-parametric test based on rank transformation (Wilcoxon's sum of rank) was used to check robustness of results under violation of condition 1- or 2- .

3- within-subject correlation

Most of the average data derives from multiple myocytes or trabeculae from different patients. We estimated within-subject correlation for each variable with One-way ANOVA. In order to account for the correlation among different cells/muscles from the same patient, we used linear mixed models[27] to compare couples of data groups, both paired and unpaired. Correction for heteroscedasticity was

---

[26] Sullivan LM, D'Agostino RB. Robustness and power of analysis of covariance applied to data distorted from normality by floor effects: homogeneous regression slopes. Statistics in medicine. 1996;15(5):477-96.

[27] Rabe-Hesketh S, Skrondal, A. and Pickles, A. Generalized multilevel structural equation modelling. Psychometrika. 2004;69 (2):167-90.

applied to linear mixed models in unpaired comparisons whenever the variances of the two groups were unequal (as calculated above by F-test).

All the results of the new statistical analysis are available on request. The Probability (*P*) values that are shown in the manuscript and in the online supplement were calculated with linear mixed models according to the aforementioned procedure.

When comparing datasets obtained with hiPSC-CMs or hiPSC-EHTs, we used one-way ANOVA with a Tukey pot-hoc test.

Statistical analysis was performed using Stata 12 software (StataCorp LP, College Station, Texas, USA).

SUPPLEMENTARY TABLES AND FIGURES

Table S1. Clinical and Instrumental Features of the four c.772G>A selected for *in vitro* studies

	ID1	ID2	ID3	ID4
Age at enrollment, yrs.	45	47	44	33
Gender	M	F	M	M
Race/ethnicity	Caucasian	Caucasian	Caucasian	Caucasian
NYHA functional class	II	I	II	II
Left atrial diameter, mm	45	48	49	51
LVOT gradient, mmHg	70	65	125	68
Maximum LV wall thickness, mm	18	21	28	23
LV End-diastolic diameter, mm	44	48	49	46
LV End-systolic diameter, mm	21	28	31	26
LV Ejection fraction, %	61	60	66	75

LV=left ventricular; LVOT=left ventricular outflow tract; NYHA=New York Heart Association. Data refer to the last visit before myectomy.

Table S2. Clinical and Instrumental Features of the 8 non-failing non-hypertrophic control patients

**Clinical / Demographic Data**	
Gender	Female 3/8 (38%)
Race/ethnicity	Caucasian 8/8 (100%)
Age at surgery	58 ± 4 yrs
Presence of known cardiomyopathies	0/8 (0%)
NYHA Class I	6/8 (75%)
NYHA Class II	2/8 (25%)
**Echo features**	
Maximal LV wall thickness	12 ± 1 mm
LA end-systolic volume	81 ± 15 mL
Ejection fraction	61 ± 4 %
LVOT gradient >30mmHg	0 /8 (0%)
Bulging septum	8/8 (100%)
**Reason for Surgery**	
Aortic valve steno-insufficiency	5/8 (63%)
Mitral valve prolapse	1/8 (12%)
Mitral valve steno-insufficiency	1/8 (12%)
Ascending aorta dilatation	1/8 (12%)

Table S3. Sequences of primers used for amplification and genotyping

Primer A*	5'-3' sequence	Primer B	3'-5' sequence	°C
D11S1785A* (6-FAM)	GCACACTGTCTTC TACAGTG (20)	D11S1785B	CTGGACCAGTAC ATTGGTAG (20)	62°
D11S4075A* (6-FAM)	TTGAGTGGACACT TGACCAC (20)	D11S4075B	AAAAGAGGACTTA CAGGGTG (20)	58°
D11S1765A* (6-FAM)	TCCAGGAAGTATC AGGACTC (20)	D11S1765B	CGGAGTTTGCACAA TCTGTC (20)	58°
D11S1344A* (HEX)	GCATCTCACCCTA AGCCTG (19)	D11S1344B	GCGCCTGGCTTGTA CATATA (20)	58°
D11S4174A* (HEX)	GCCCACTATGTAG CTTTTCC (20)	D11S4174B	CCCAGATGGTTACA TGACTG (20)	60°
D11S1313A* (HEX)	TCCAGTGTCAAAGT ACCCAG (20)	D11S1313B	CCAACGTCTAAGCA TGAAGC (20)	60°
MYBPC3_CA _1_A* (6-FAM)	GAGCTTGTCCCCTT CCAAG (19)	MYBPC3_CA _1_B	TGCTCTGAGGTGTT GAAAGC (20)	62°
MYBPC3_CA _2_A* (HEX)	GCAAAGGCCTGTC TTAACTC (20)	MYBPC3_CA _2_B	GAGAAGGCAGCTCT GTATTC (20)	60°
MYBPC3_GT _1_A* (6-FAM)	AATGCAGCCCCTCTT TTCTC (20)	MYBPC3_GT _1_B	TCTCCTGGACTCTTG AAACC (20)	60°
MYBPC3_CA _3_A* (HEX)	TGCAACTTGAAGTCT CTGCC (20)	MYBPC3_CA _3_B	CCCAGGTATGTATAC TGGAG (20)	60°
MYBPC3_CA _4_A* (6-FAM)	TGAGACATGATGAG AGGTTCC (21)	MYBPC3_CA _4_B	CTCTGGTTTCCACAT CTCTG (20)	60°

Myofibril type	RT	Po	k_{ACT}	k_{TR}	D_{slow}	slow k_{REL}	fast k_{REL}
	kN m^{-2}	kN m^{-2}	s^{-1}	s^{-1}	ms	s^{-1}	s^{-1}
Donors[1] (N=5)	10.4±0.6 (n=98)	111±5 (n=96)	0.85±0.02 (n=118)	0.71±0.02 (n=102)	186±5 (n=100)	0.29±0.02 (n=95)	4.18±0.1 (n=101)
c.772G>A (N=3)	12.3±1.2 (n=49)	111.4±7 (n=51)	1.15±0.06* (n=50)	1.03±0.08* (n=48)	155±6.3* (n=42)	0.65±0.05** (n=41)	6.4±0.4** (n=50)

Table S4. Mechanical and kinetic parameters of myofibrils from c.772G>A HCM and donor patients. [1]Data from Piroddi *et al.* (2019). Data are Means ± SE; N, number of patients in the group; n, number of myofibrils; RT, resting tension; Po, maximal Ca^{2+} -activated tension; D_{slow}, slow phase duration. *P < 0.05; **P< 0.01.

Ion current or parameter	Scaling factor
INaL	2.4
INab	2.65
Ito	0.3
IKr	0.55
IKs	0.55
IK1	0.7
INaCa	1.3
INaK	0.7
Jup	0.85
Jrel	0.8
koff	0.8
tau ICaL *fast inactivation*	1.35
tau ICaL *slow inactivation*	1.2
Cell radius	$\sqrt{1.9}$

Table S5. HCM ionic remodeling from Coppini et al.,2013 and Passini et al., 2016.

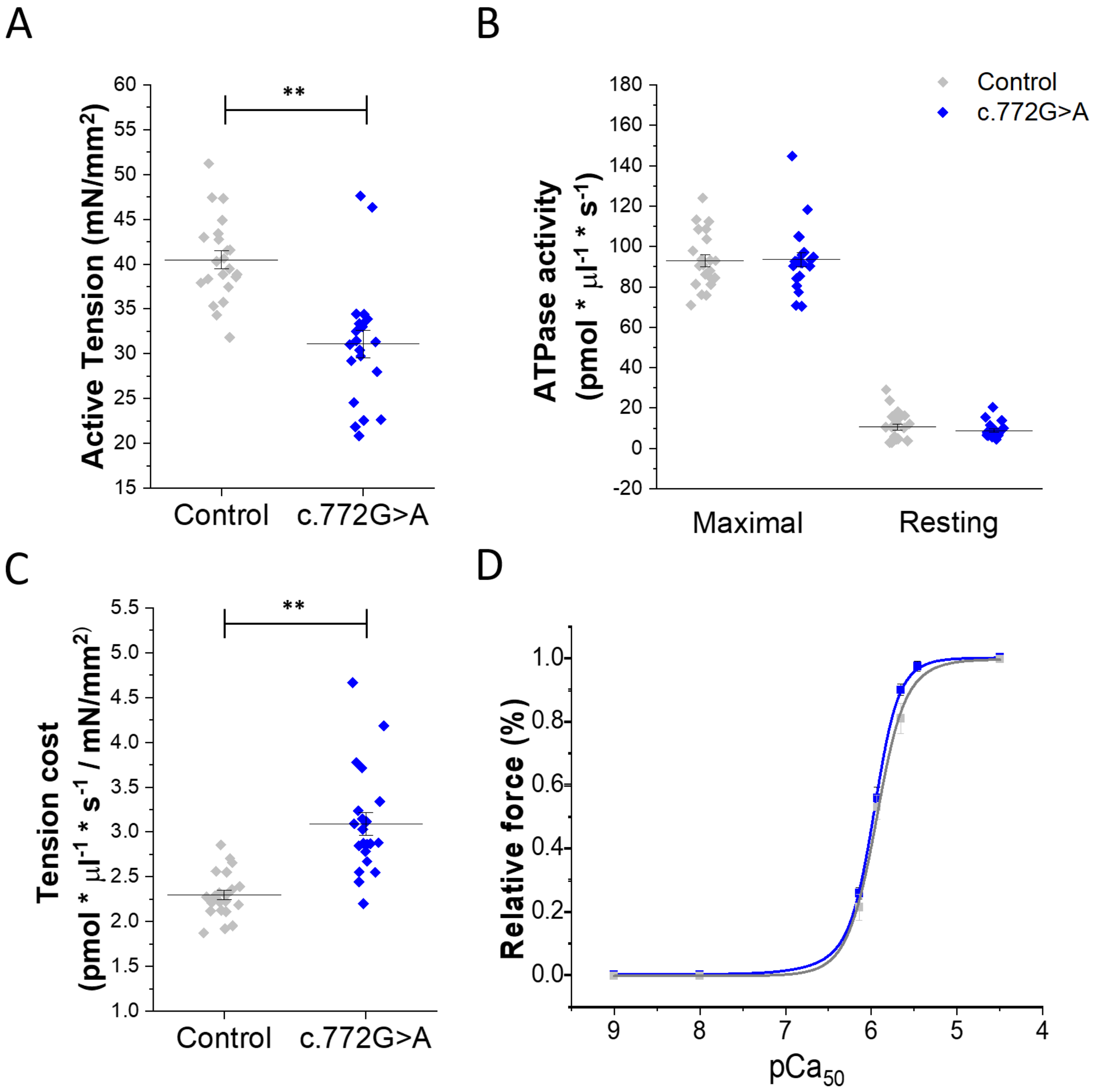

Figure S1. Effect of the c.772G>A mutation on tension cost and Ca^{2+}-sensitivity. (**A**) Maximal active tension in skinned ventricular strips from donors and c.772G>A. (**B**) Maximal Ca^{2+}-activated ATPase and basal ATPase activities in c.772G>A and donor muscle strips. (**C**) Tension cost determined as the ratio between maximal Ca^{2+}-activated ATPase and maximal active tension in c.772G>A and donor muscle strips. (**A-C**) data from *n*=13 strips obtained from *N*=3 c.772G>A patient samples; control data from *n*=13 strips from *N*=2 donor samples. (**D**) Mean pCa-active tension curve and mean values of pCa at half-maximal activation (pCa_{50}) from donors (n=18) and c.772G>A (N=3, n=19). (**A-D**) Lines and error bars in the scatter plots represent the mean ± SEM. Statistics: hierarchical statistical analysis was used to compare all c.772G>A datasets vs. controls. P values were calculated using linear mixed models. *=P<0.05, **=P<0.01. *N*=number of patients, *n*=number of individual muscle strips

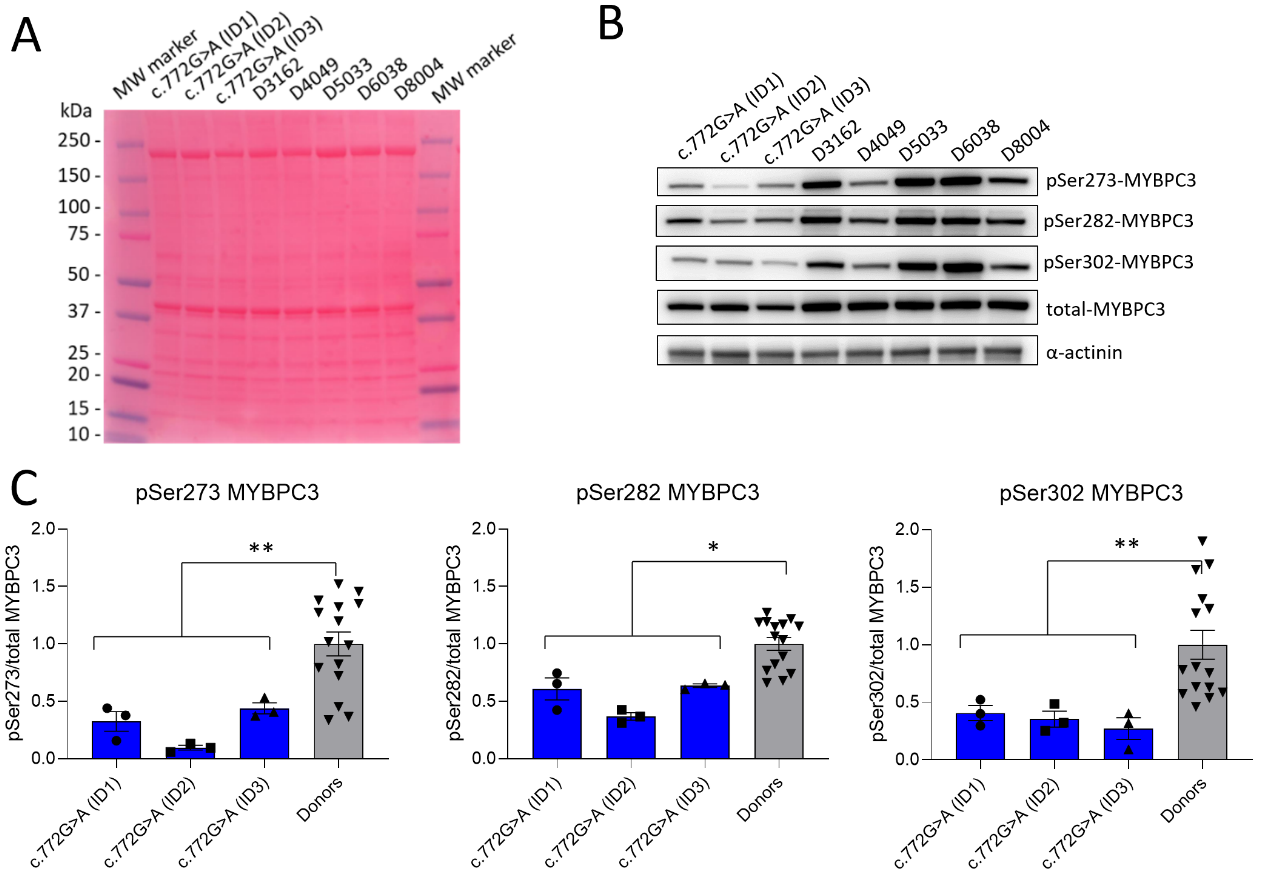

Figure S2. Analyses of cMyBP-C protein phosphorylation at specific phospho-sites. (**A**) Ponceau-stained protein blots of HCM and donor samples (numbers and codes refer to the individual samples). Five control samples were included (donors 3162, 4049, 5033, 6038 and 8014). (**B**) Blots were stained with specific antibodies against cMyBP-C, and specific phosphorylation sites Ser273, Ser 282 and Ser302. Panel B shows representative blots. The full uncut blots are shown in Fig S7. (**C**) Samples were run 6 times for the analysis of cMyBP-C/α-actinin, and 3 times for analyses of phosphorylation levels at the 3 phospho-sites relative to total cMyBP-C. Reduced levels of full-length cMyBP-C (140 kDa) normalized to α-actinin was observed in cardiac biopsies from c.772G>A patients compared with donor samples. In addition, the level of cMyBP-C phosphorylation at the 3 phospho-sites normalized to total cMyBP-C was lower in the c.772G>A samples compared to controls. Donors N=5, c.772G>A N=3. Statistics: p values were calculated using linear mixed models as described above. *=P<0.05; **=P<0.01

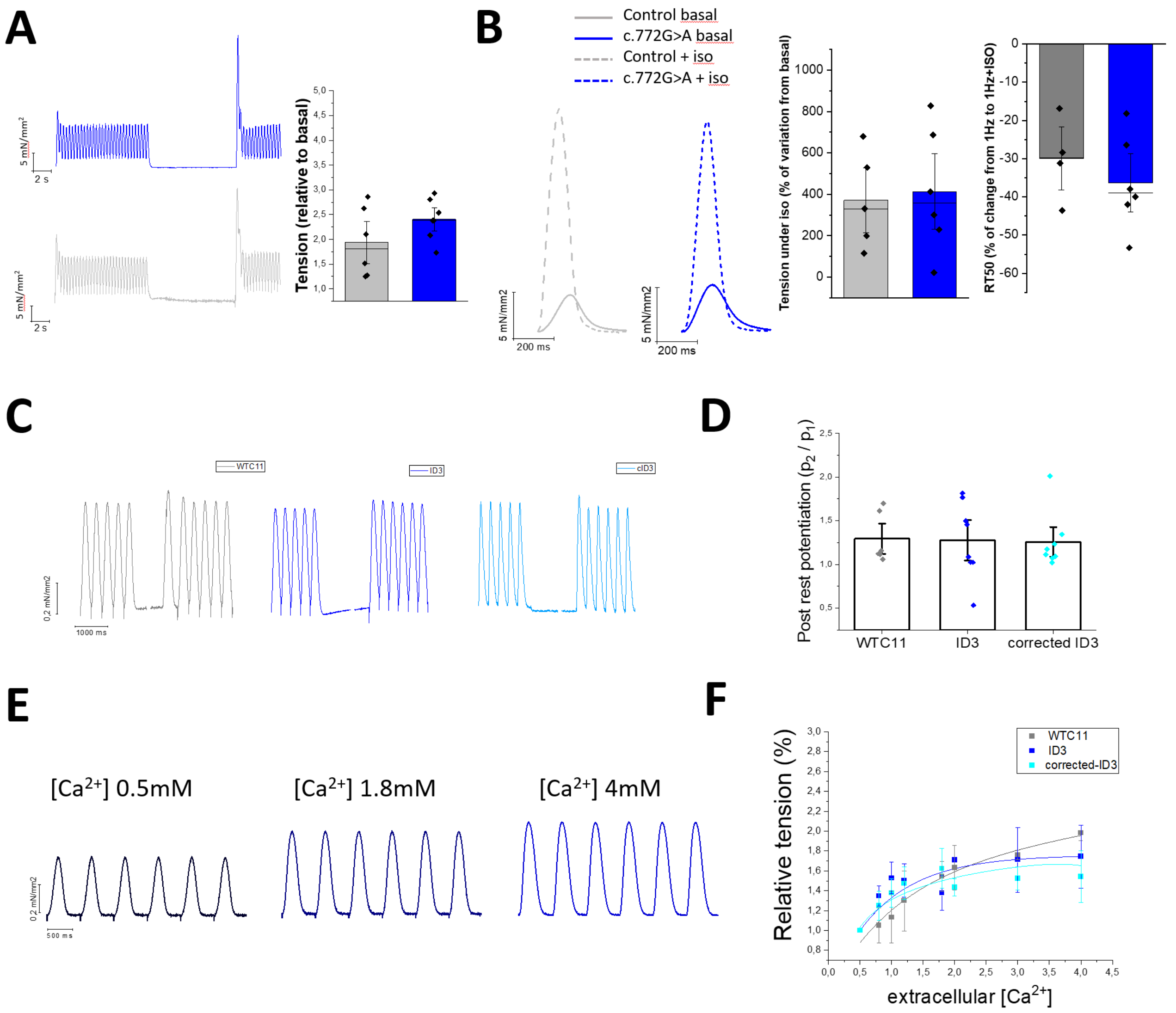

Figure S3. Inotropic interventions on intact trabeculae and engineered heart tissues (EHTs). **(A)** Left: Representative continuous recording from a c.772G>A and a control trabecula during a post-rest protocol. Right: Positive inotropic responses to long pauses. **(B)** Left: Superimposed twitches from an c.772G>A and control trabeculae at baseline and in the presence of 0.1 µmol/L isoproterenol (Iso). Right: average percentage variation of force amplitude and twitch duration to 50% relaxation in control and c.772G>A intact trabeculae upon addition of β-adrenergic stimulation with isoproterenol 10^{-6}M; c.772G>A: n=4, N=2; control: n=5, N=4. (**A-B**) Statistics: hierarchical statistical analysis was used to compare all c.772G>A datasets vs. controls. P values were calculated using linear mixed models. No significant differences were observed. N=number of patients, n=number of individual muscle strips **(C)** Representative continuous recording from an ID3-(c.772G>A), corrected-ID3 and control WTC11 hiPSC-EHTs during a post-rest protocol. **(D)** Positive inotropic responses to pauses in EHTs from the 3 cell lines. **(E)** Superimposed twitches from EHTs at different calcium concentration (0.5 to 4 mM). **(F)** Fitting curves of the percentage of variation in force amplitude measured in EHTs upon changes of extracellular calcium concentration from 0.5 to 4mM. ID3 (N=7; n=12) were compared with the corrected isogenic control (corrected ID3, N=5, n=7) and the healthy control (WTC11, N=3, n=8). (**D,F**) Statistical analysis was performed using one-way ANOVA with Tukey post-hoc test. No statistically significant differences emerged.

A

	Pluripotency Markers			
	Oct4	Nanog	SSEA-4	TRA-1-60
**Cell Line**				
WTC11	98.2%	90.8%	99.9%	97.6%
Clone 1 ID3	99.1%	95.5%	100.0%	91.6%
Corrected ID3	99.6%	97.7%	99.9%	99.8%

B

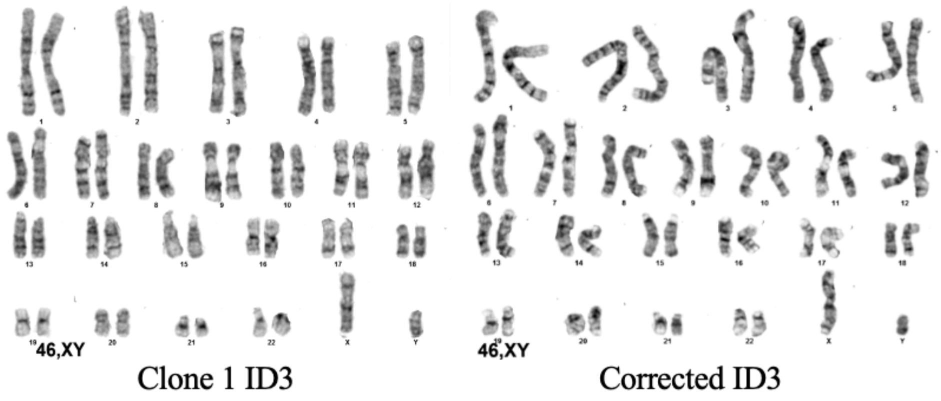

C

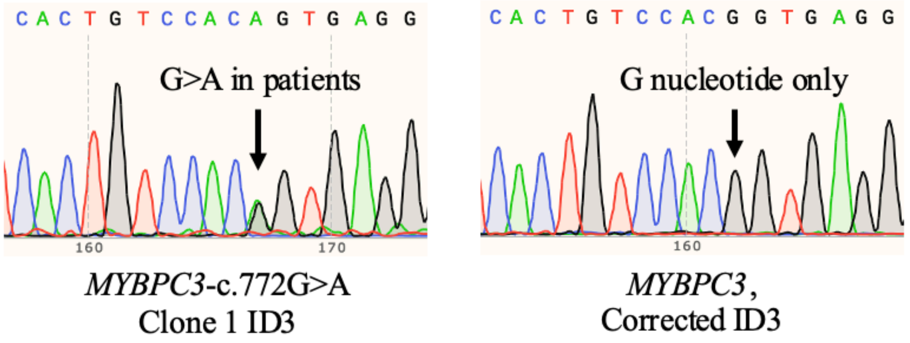

Figure S4. Quality control characterization of ID3 and corrected ID3 patient hiPSC lines. (**A**) Oct4, Nanog, SSEA-4, and TRA-1-60 pluripotency markers quantified by flow cytometry, (**B**) healthy karyotyping, and (**C**) confirmed presence of the *MYBPC3:*c.772G>A nucleotide switch in the patient ID3 line and restoration of the normal genotype in the corrected ID3 line.

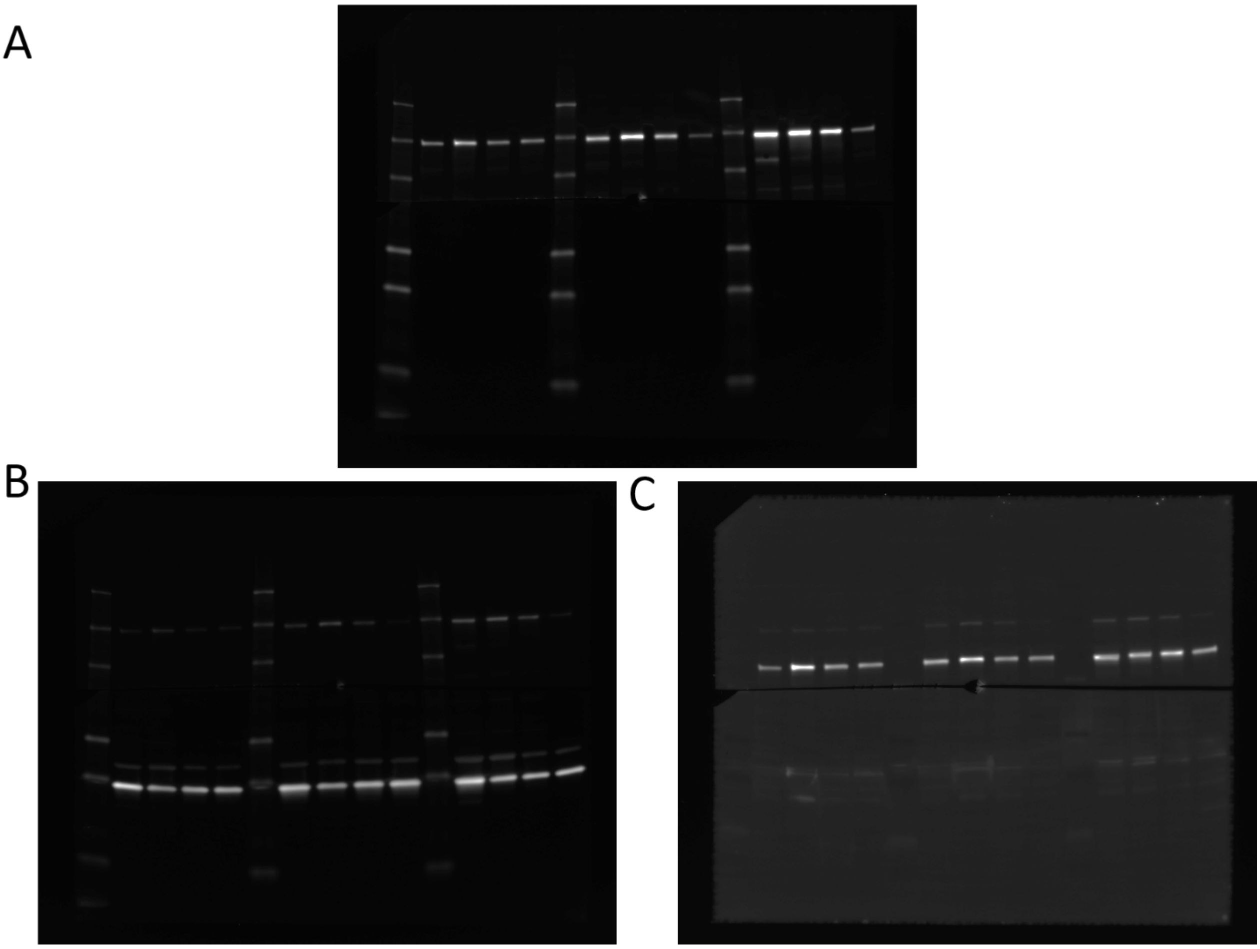

Figure S5. Full uncut western blots for (**A**) cMyBP-C, (**B**) GAPDH, and (**C**) α-actinin. Average results are in Main **Fig 7 panel B**. Lanes 1-15 (left to right): ladder, WTC11 day 14, WTC11 day 30, WTC11 day 45, WTC11 day 60, ladder, ID3 day 14, ID3 day 30, ID3 day 45, ID3 day 60, ladder, corrected ID3 day 14, corrected ID3 day 30, corrected ID3 day 45, corrected ID3 day 60.

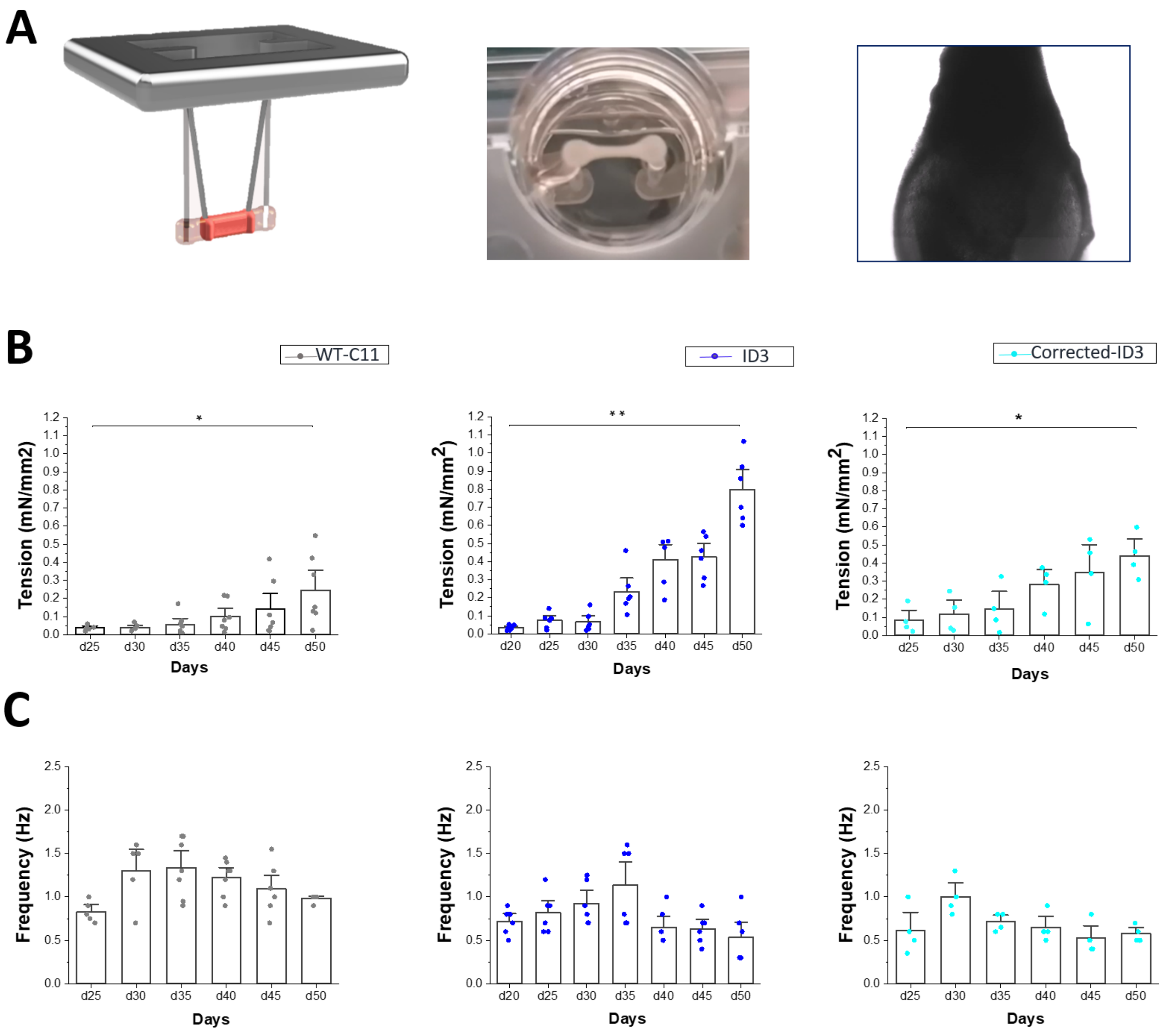

Figure S6. Long-term spontaneous auxotonic contractions of engineered heart tissues (EHTs). (**A**) Left: 3D render of silicon posts and EHTs (figure art by Marco Catarzi). Center: representative image of an engineered heart tissue (bottom view, 4x magnification) formed between two calibrated silicone posts. Right: bottom view of a single post (10x). (**B**) Spontaneous auxotonic twitch tension of EHTs from day 25 to 50 of differentiation, measured at 37°C in RPMI/B27 culture medium with 5% CO_2 (~0.4 mM of external Ca^{2+}). (**C**) Progression of spontaneous beating frequency of all EHTs from day 20 to 50. WTC11: n=7, ID3: n=6 and corrected ID3: n=4. One-way analysis of variance (ANOVA) with a Tukey post-hoc test was used to compare the different time points. * $p < 0.05$ and ** $p < 0.01$ versus d25.

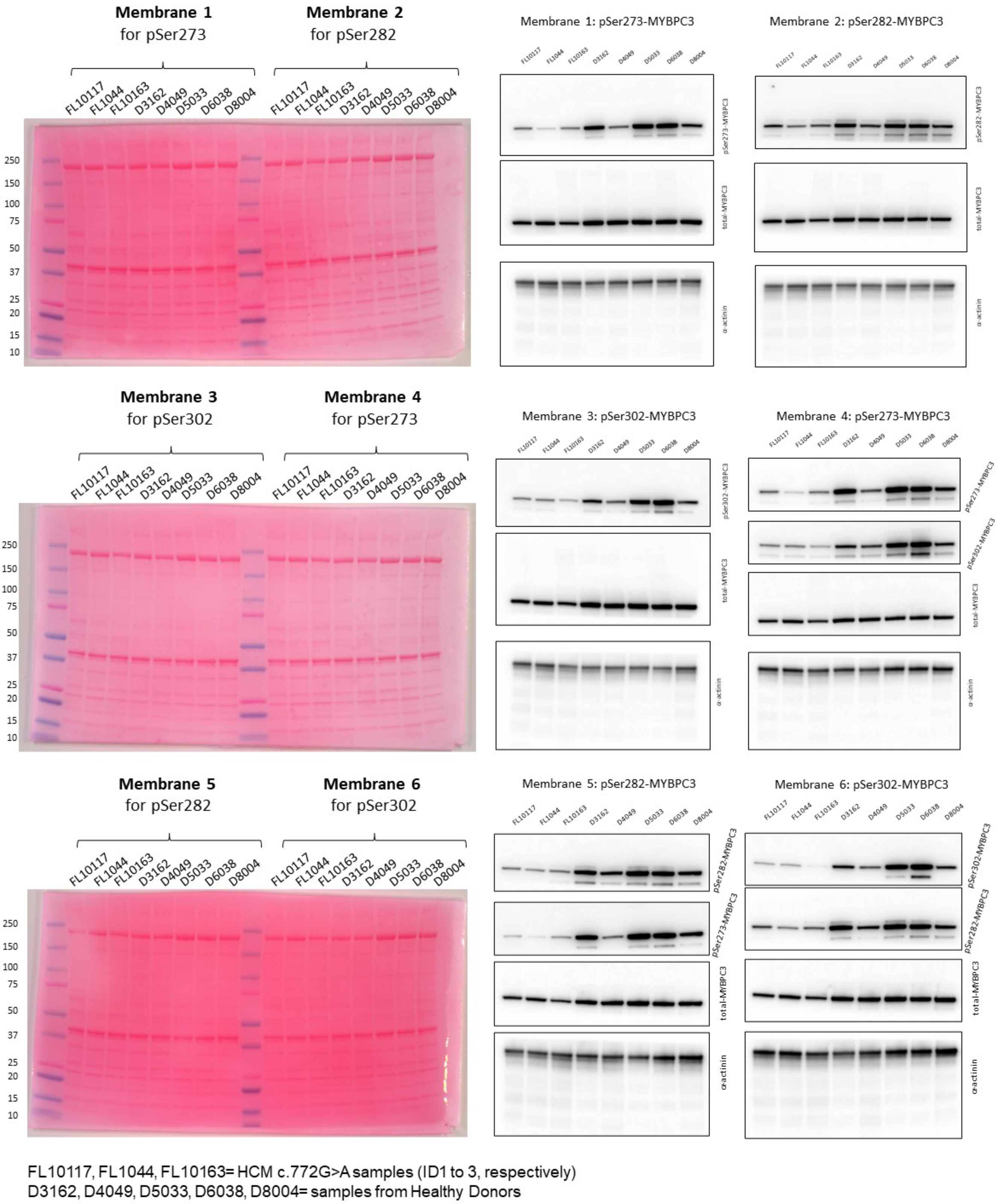

Figure S7. Uncropped Ponceau stains and western blots. Figure shows all the uncut blots related to Main Figure 7 panel A and Figure S2.

SELECTED EXAMPLES OF STATISTICAL ANALYSIS

The following examples report the statistical procedure and the output obtained from the Stata 12.0 program (text in blue).

EXAMPLE 1: Tension Cost in muscle strips (Figure S1 panel C)

Variable name= "tcost"
caco=1 : control subjects
caco=2 : HCM subjects
subj : individual patients

- STEP 1) non-Gaussian distribution

The data were tested for normality using the Skewness/Kurtosis test

```
. . sktest tcost

                    Skewness/Kurtosis tests for Normality
                                                  ------- joint ------
    Variable |    Obs   Pr(Skewness)   Pr(Kurtosis)  adj chi2(2)    Prob>chi2
-------------+--------------------------------------------------------------
       tcost |         35      0.0052           0.0773           9.15        0.0103

. . sktest tcost if caco==1

                    Skewness/Kurtosis tests for Normality
                                                  ------- joint ------
    Variable |    Obs   Pr(Skewness)   Pr(Kurtosis)  adj chi2(2)    Prob>chi2
-------------+--------------------------------------------------------------
       tcost |     14              0.1223        0.1129        4.90            0.0864

. . sktest tcost if caco==2

                    Skewness/Kurtosis tests for Normality
                                                  ------- joint ------
    Variable | Obs   Pr(Skewness)  Pr(Kurtosis)  adj chi2(2)  Prob>chi2
-------------+--------------------------------------------------------------
       tcost |    21      0.0223           0.1351          6.69            0.0352
```

STEP 2) Heteroschedasticy (unequality of variances)

We used the F test for equality of variances in two-group comparison studies

```
. . sdtest tcost, by(caco)

Variance ratio test
------------------------------------------------------------------------------
   Group |  Obs       Mean     Std. Err.   Std. Dev.   [95% Conf. Interval]
---------+--------------------------------------------------------------------
       1 |      14    2.275714    .0632865    .2367964    2.138992    2.412436
       2 |      21    3.088571    .1289468    .5909085    2.819593     3.35755
---------+--------------------------------------------------------------------
combined | 35  2.763429     .10557    .6245608      2.548884    2.977973
------------------------------------------------------------------------------
    ratio = sd(1) / sd(2)                                   f =   0.1606
```

```
Ho: ratio = 1                          degrees of freedom =    13, 20

    Ha: ratio < 1             Ha: ratio != 1              Ha: ratio > 1
  Pr(F < f) = 0.0008         2*Pr(F < f) = 0.0016          Pr(F > f) = 0.9992
```

RESULTS OF STEP 1 AND 2: Although overall datapoints are normally distributed, the distribution is not normal in control subjects. Moreover, the variances in HCM and control samples were different. Therefore, non-parametric test based on rank transformation (Wilcoxon's sum of rank and Kruskal-Wallis) will be used at the end to check robustness of results under violation of condition 1- or 2- .

```
. . ranksum tcost, by(caco)

Two-sample Wilcoxon rank-sum (Mann-Whitney) test

        caco |      obs    rank sum    expected
-------------+---------------------------------
           1 |       14         122         252
           2 |       21         508         378
-------------+---------------------------------
    combined |       35         630         630

unadjusted variance      882.00
adjustment for ties       -0.49
                     ----------
adjusted variance        881.51

Ho: tcost(caco==1) = tcost(caco==2)
             z =  -4.379
    Prob > |z| =   0.0000
```

The robustness of the results is confirmed.

- STEP 3) Within-subject correlation

We first tested whether inter-subject variability is higher that the variability among myofibrils from the same subject, using One-Way ANOVA

```
. . loneway tcost subj

                   One-way Analysis of Variance for tcost:

                                         Number of obs =        35
                                             R-squared =    0.4390

    Source                  SS         df      MS              F     Prob > F
-------------------------------------------------------------------------
Between subj          5.8226845      4   1.4556711      5.87     0.0013
Within subj          7.4399043      30   .24799681
-------------------------------------------------------------------------
Total               13.262589     34   .39007614

         Intraclass       Asy.
         correlation      S.E.       [95% Conf. Interval]
         ------------------------------------------------
           0.41526     0.22464       0.00000     0.85554
```

```
Estimated SD of subj effect          .4196655
Estimated SD within subj             .4979928
Est. reliability of a subj mean      0.82963
     (evaluated at n=6.86)
```

Inter-subject variability is higher

Therefore, we used linear mixed models to account for within-subject correlation. Due to inequality of variances, we adjusted the model for heteroschedasticity.

```
. . xtmixed tcost caco || subj: , residuals(independent, by(caco))

Obtaining starting values by EM:

Performing gradient-based optimization:

Iteration 0:   log likelihood = -23.315818
Iteration 1:   log likelihood =   -17.5319
Iteration 2:   log likelihood = -17.423419
Iteration 3:   log likelihood = -17.416056
Iteration 4:   log likelihood = -17.416054

Computing standard errors:

Mixed-effects ML regression                     Number of obs      =        35
Group variable: subj                            Number of groups   =         5

                                                Obs per group: min =         4
                                                               avg =       7.0
                                                               max =        10


                                                Wald chi2(1)       =     33.79
Log likelihood = -17.416054                     Prob > chi2        =    0.0000

------------------------------------------------------------------------------
       tcost |      Coef.   Std. Err.      z    P>|z|     [95% Conf. Interval]
-------------+----------------------------------------------------------------
        caco |   .8128571   .1398378     5.81   0.000     .5387802    1.086934
       _cons |   1.462857   .1752481     8.35   0.000     1.119377    1.806337
------------------------------------------------------------------------------


------------------------------------------------------------------------------
  Random-effects Parameters  |   Estimate   Std. Err.     [95% Conf. Interval]
-----------------------------+------------------------------------------------
subj: Identity               |
                   sd(_cons) |   1.13e-08          .             .          .
-----------------------------+------------------------------------------------
Residual: Independent,       |
    by caco                  |
                  1: sd(e)   |   .2281827   .0431225      .1575508    .3304796
                  2: sd(e)   |   .5766677   .0889818      .4261695     .780313
------------------------------------------------------------------------------
LR test vs. linear regression:       chi2(2) =    11.56   Prob > chi2 = 0.0031
```

As mentioned before, we here use Wilcoxon's sum of rank to check robustness of the identified difference

```
. . ranksum tcost, by(caco)

Two-sample Wilcoxon rank-sum (Mann-Whitney) test

        caco |      obs    rank sum    expected
-------------+---------------------------------
           1 |       14         122         252
           2 |       21         508         378
-------------+---------------------------------
    combined |       35         630         630

unadjusted variance       882.00
adjustment for ties        -0.49
                      ----------
adjusted variance         881.51

Ho: tcost(caco==1) = tcost(caco==2)
             z =  -4.379
    Prob > |z| =   0.0000
```

The robustness of the results is confirmed.

The statistical analysis demonstrated a significant difference between tension cost in control and HCM samples.

EXAMPLE 2: maximal force (p0) in myofibrils (Fig 4, panel D, first graph)

Variable name= "p0"
caco=1 : control subjects
caco=2 : HCM subjects
subj : individual patients

- STEP 1) non-Gaussian distribution

The data were tested for normality using the Skewness/Kurtosis test

```
. . sktest p0

                   Skewness/Kurtosis tests for Normality
                                                     ------- joint ------
    Variable |    Obs   Pr(Skewness)   Pr(Kurtosis)  adj chi2(2)    Prob>chi2
-------------+---------------------------------------------------------------
          p0 |    147     0.0004          0.2887         11.74        0.0028

. . sktest p0 if caco==1

                   Skewness/Kurtosis tests for Normality
                                                     ------- joint ------
    Variable |    Obs   Pr(Skewness)   Pr(Kurtosis)  adj chi2(2)    Prob>chi2
-------------+---------------------------------------------------------------
          p0 |     96     0.0003          0.0987         12.80        0.0017
```

```
. . sktest p0 if caco==2

                   Skewness/Kurtosis tests for Normality
                                                  ------- joint ------
    Variable |    Obs   Pr(Skewness)   Pr(Kurtosis)  adj chi2(2)    Prob>chi2
-------------+---------------------------------------------------------------
          p0 |     51      0.2306         0.5264          1.93         0.3806
```

- STEP 2) Heteroschedasticy (unequality of variances)

```
. . sdtest p0, by(caco)

Variance ratio test
------------------------------------------------------------------------------
   Group | Obs     Mean       Std. Err.     Std. Dev.   [95% Conf. Interval]
---------+--------------------------------------------------------------------
       1 |      96     111.294     5.38136    52.72634    100.6106    121.9773
       2 |      51    111.4483    7.427799     53.0451    96.52916    126.3675
---------+--------------------------------------------------------------------
combined |147  111.3475    4.342933    52.65527  102.7644    119.9307
------------------------------------------------------------------------------
    ratio = sd(1) / sd(2)                                f =   0.9880
Ho: ratio = 1                            degrees of freedom =    95, 50

    Ha: ratio < 1              Ha: ratio != 1                 Ha: ratio > 1
  Pr(F < f) = 0.4704         2*Pr(F < f) = 0.9408          Pr(F > f) = 0.5296
```

Variances are EQUAL.

- STEP 3) Within-subject correlation

We first tested whether inter-subject variability is higher that the variability among myofibrils from the same subject, using One-Way ANOVA

```
. . loneway p0 subj

                  One-way Analysis of Variance for p0:

                                   Number of obs =       146
                                      R-squared =     0.1083

    Source               SS                df        MS              F       Prob > F
-------------------------------------------------------------------------
Between subj        43573.974       6      7262.3289      2.81     0.0130
Within subj         358923.98     139     2582.1869
-------------------------------------------------------------------------
Total                402497.96     145     2775.848

         Intraclass       Asy.
         correlation       S.E.       [95% Conf. Interval]
         ------------------------------------------------
           0.08240      0.07082       0.00000      0.22122

         Estimated SD of subj effect            15.22791
         Estimated SD within subj               50.81522
         Est. reliability of a subj mean        0.64444
              (evaluated at n=20.18)
```

Inter-subject variability is higher

Therefore, we used linear mixed models to account for within-subject correlation.

```
. . xtmixed p0 caco || subj:

Performing EM optimization:

Performing gradient-based optimization:

Iteration 0:   log likelihood = -783.84759
Iteration 1:   log likelihood = -783.84759

Computing standard errors:

Mixed-effects ML regression              Number of obs      =       146
Group variable: subj                     Number of groups   =         7

                                         Obs per group: min =        15
                                                        avg =      20.9
                                                        max =        43


                                         Wald chi2(1)       =      0.06
Log likelihood = -783.84759              Prob > chi2        =    0.8134

------------------------------------------------------------------------------
          p0 |      Coef.   Std. Err.      z    P>|z|     [95% Conf. Interval]
-------------+----------------------------------------------------------------
        caco |   3.230972   13.69133     0.24   0.813    -23.60354    30.06549
       _cons |   104.4942   20.36695     5.13   0.000     64.57571    144.4127
------------------------------------------------------------------------------

------------------------------------------------------------------------------
  Random-effects Par   |   Estimate   Std. Err.     [95% Conf. Interval]
-----------------------------+------------------------------------------------
subj: Identity               |
                   sd(_cons) |   13.47575   6.434974      5.285489    34.35744
-----------------------------+------------------------------------------------
                sd(Residual) |   50.85076   3.053136      45.2054    57.20112
------------------------------------------------------------------------------
LR test vs. linear regression: chibar2(01) =     3.22 Prob >= chibar2 = 0.0364
```

The results of step 3 confirmed that p0 is not significantly different between HCM and control myofibrils.

EXAMPLE 3: decay time-constant of linear slow phase of myofibril relation (slowKrel), figure 4 panel D (fifth graph from left)

Variable name= “slowKrel”
caco=1 : control subjects
caco=2 : HCM subjects
subj : individual patients

- STEP 1) non-Gaussian distribution

The data were tested for normality using the Skewness/Kurtosis test

```
. . sktest slowKrel

                    Skewness/Kurtosis tests for Normality
                                                    ------- joint ------
    Variable |    Obs   Pr(Skewness)   Pr(Kurtosis)  adj chi2(2)    Prob>chi2
-------------+---------------------------------------------------------------
    slowKrel |    136      0.0000          0.0000         44.55         0.0000

. . sktest slowKrel if caco==1

                    Skewness/Kurtosis tests for Normality
                                                    ------- joint ------
    Variable |    Obs   Pr(Skewness)   Pr(Kurtosis)  adj chi2(2)    Prob>chi2
-------------+---------------------------------------------------------------
    slowKrel |     92      0.0000          0.0005         22.94         0.0000

. . sktest slowKrel if caco==2

                    Skewness/Kurtosis tests for Normality
                                                    ------- joint ------
    Variable |    Obs   Pr(Skewness)   Pr(Kurtosis)  adj chi2(2)    Prob>chi2
-------------+---------------------------------------------------------------
    slowKrel |     41      0.0012          0.0327         12.07         0.0024
```

Data is normally distributed

- STEP 2) Heteroschedasticy (unequality of variances)

```
. . sdtest slowKrel, by(caco)

Variance ratio test
------------------------------------------------------------------------------
   Group |Obs      Mean      Std. Err.    Std. Dev.   [95% Conf. Interval]
---------+--------------------------------------------------------------------
       1 |     92    .2986413    .0165603    .1588404    .2657464    .3315362
       2 |     41    .6497073    .0485408    .3108129    .5516027    .747812
---------+--------------------------------------------------------------------
combined |133   .4068647   .0234518    .270459    .3604747    .4532546
------------------------------------------------------------------------------
    ratio = sd(1) / sd(2)                                  f =   0.2612
Ho: ratio = 1                             degrees of freedom =    91, 40

    Ha: ratio < 1               Ha: ratio != 1                 Ha: ratio > 1
  Pr(F < f) = 0.0000         2*Pr(F < f) = 0.0000           Pr(F > f) = 1.0000
```

Variances are largely different

RESULTS OF STEP 1 AND 2: Although overall datapoints are normally distributed, the variances in HCM and control samples were different. Therefore, non-parametric test based on rank transformation (Wilcoxon's sum of rank and Kruskal-Wallis) were used to check robustness of results under violation of condition 1- or 2- .

```
. . ranksum slowKrel, by(caco)
```

Two-sample Wilcoxon rank-sum (Mann-Whitney) test

```
        caco |      obs    rank sum    expected
-------------+---------------------------------
           1 |       92      4742.5        6164
           2 |       41      4168.5        2747
-------------+---------------------------------
    combined |      133        8911        8911

unadjusted variance    42120.67
adjustment for ties      -16.11
                     ----------
adjusted variance      42104.55

Ho: slowKrel(caco==1) = slowKrel(caco==2)
             z =  -6.928
    Prob > |z| =   0.0000
```

The robustness of the results is confirmed

- STEP 3) Within-subject correlation

We first tested whether inter-subject variability is higher that the variability among myofibrils from the same subject, using One-Way ANOVA

```
. . loneway slowKrel subj

                  One-way Analysis of Variance for slowKrel:

                                              Number of obs =        132
                                                  R-squared =     0.4131

    Source                SS         df      MS            F     Prob > F
-------------------------------------------------------------------------
Between subj          3.9808796      7    .56869709     12.47     0.0000
Within subj           5.6557942    124    .04561124
-------------------------------------------------------------------------
Total                 9.6366738    131    .0735624

         Intraclass       Asy.        
         correlation      S.E.       [95% Conf. Interval]
         ------------------------------------------------
            0.41943     0.15619       0.11330     0.72556

         Estimated SD of subj effect             .1815252
         Estimated SD within subj                .2135679
         Est. reliability of a subj mean          0.91980
              (evaluated at n=15.87)
```

Inter-subject variability is higher

Therefore, we used linear mixed models to account for within-subject correlation. Due to inequality of variances, we adjusted the model for heteroschedasticity.

```
. . xtmixed slowKrel caco ||  subj: , residuals(independent, by(caco))

Obtaining starting values by EM:
```

```
Performing gradient-based optimization:

Iteration 0:   log likelihood =  15.285201
Iteration 1:   log likelihood =  29.081895
Iteration 2:   log likelihood =  29.593658
Iteration 3:   log likelihood =  29.594104
Iteration 4:   log likelihood =  29.594104

Computing standard errors:

Mixed-effects ML regression                 Number of obs      =       132
Group variable: subj                    Number of groups   =         8

                                        Obs per group: min =         7
                                                       avg =      16.5
                                                       max =        37


                                        Wald chi2(1)       =     37.01
Log likelihood =  29.594104                 Prob > chi2        =    0.0000

------------------------------------------------------------------------------
    slowKrel |      Coef.   Std. Err.      z    P>|z|     [95% Conf. Interval]
-------------+----------------------------------------------------------------
        caco |   .3527253   .0579822     6.08   0.000     .2390822    .4663684
       _cons |  -.0590279   .0715259    -0.83   0.409    -.1992162    .0811603
------------------------------------------------------------------------------

------------------------------------------------------------------------------
  Random-effects Param|  Estimate   Std. Err.     [95% Conf. Interval]
-----------------------------+------------------------------------------------
subj: Identity               |
                   sd(_cons) |   .0377719   .0211344      .0126154    .1130937
-----------------------------+------------------------------------------------
Residual: Independent,       |
    by caco                  |
                    1: sd(e) |   .1538381   .0116847      .1325596    .1785323
                    2: sd(e) |   .3056638   .0340762      .2456687    .3803103
------------------------------------------------------------------------------
LR test vs. linear regression:       chi2(2) =    29.23   Prob > chi2 = 0.0000
```

The statistical analysis demonstrated a significant difference between slowKrel in control and HCM myofibrils.

EXAMPLE 4: AP duration at 90% repolarization (APD90) at 1Hz in human myocytes (Fig. 5B, left part).

Variable name= “apd90”
caco=1 : control subjects
caco=2 : HCM subjects
subj : individual patients

- STEP 1) non-Gaussian distribution

The data were tested for normality using the Skewness/Kurtosis test

```
. . sktest apd90
Skewness/Kurtosis tests for Normality
                                            ------- joint ------
    Variable |    Obs   Pr(Skewness)   Pr(Kurtosis)  adj chi2(2)    Prob>chi2
-------------+----------------------------------------------------------------
       apd90 |     32      0.1475         0.5076         2.76         0.2518
```

Data is not normally distributed

- STEP 2) Heteroschedasticy (unequality of variances)

```
sdtest apd90, by(caco)

Variance ratio test
------------------------------------------------------------------------------
   Group |     Obs        Mean    Std. Err.   Std. Dev.   [95% Conf. Interval]
---------+--------------------------------------------------------------------
       1 |      21    397.8095    16.49626    75.59538    363.3989    432.2201
       2 |      11         677     28.6439    95.00105    613.1774    740.8226
---------+--------------------------------------------------------------------
combined |      32    493.7813    27.81034     157.319    437.0617    550.5008
------------------------------------------------------------------------------
    ratio = sd(1) / sd(2)                                         f =   0.6332
Ho: ratio = 1                                    degrees of freedom =   20, 10

    Ha: ratio < 1               Ha: ratio != 1                 Ha: ratio > 1
  Pr(F < f) = 0.1843         2*Pr(F < f) = 0.3686           Pr(F > f) = 0.8157
```

Variances are equal

RESULTS OF STEP 1 AND 2: Although the variances in HCM and control samples were equal, overall datapoints are not normally distributed. Therefore, non-parametric test based on rank transformation (Wilcoxon's sum of rank and Kruskal-Wallis) will be used at the end to check robustness of results under violation of condition 1- or 2- .

- STEP 3) Within-subject correlation

We first tested whether inter-subject variability is higher that the variability among myocytes from the same subject, using One-Way ANOVA

```
loneway apd90 subj

                  One-way Analysis of Variance for apd90:
                                         Number of obs =        32
                                             R-squared =    0.7610

    Source                SS         df      MS            F     Prob > F
-------------------------------------------------------------------------
Between subj          583877.64      9   64875.293      7.78     0.0000
Within subj           183349.83     22   8334.0833
-------------------------------------------------------------------------
Total                 767227.47     31   24749.273

         Intraclass       Asy.
         correlation      S.E.       [95% Conf. Interval]
         ------------------------------------------------
            0.68082     0.14141       0.40367     0.95798
```

```
        Estimated SD of subj effect          133.331
        Estimated SD within subj             91.2912
        Est. reliability of a subj mean      0.87154
             (evaluated at n=3.18)
```

Inter-subject variability is higher

Therefore, we used linear mixed models to account for within-subject correlation.

```
. xtmixed apd90 caco ||  subj:

Performing EM optimization:

Performing gradient-based optimization:

Iteration 0:   log likelihood = -185.84714
Iteration 1:   log likelihood = -185.61242
Iteration 2:   log likelihood = -185.61097
Iteration 3:   log likelihood = -185.61097

Computing standard errors:

Mixed-effects ML regression                 Number of obs      =        32
Group variable: subj                        Number of groups   =        10

                                      Obs per group: min =         2
                                                     avg =       3.2
                                                     max =         4


                                      Wald chi2(1)       =     88.03
Log likelihood = -185.61097                  Prob > chi2        =    0.0000

------------------------------------------------------------------------------
       apd90 |      Coef.   Std. Err.      z    P>|z|     [95% Conf. Interval]
-------------+----------------------------------------------------------------
        caco |   279.1905   29.75697     9.38   0.000     220.8679    337.5131
       _cons |    118.619   42.41021     2.80   0.005     35.49657    201.7415
------------------------------------------------------------------------------


------------------------------------------------------------------------------
  Random-effects Parameters  |   Estimate   Std. Err.     [95% Conf. Interval]
-----------------------------+------------------------------------------------
subj: Identity               |
                   sd(_cons) |   1.57e-10   1.02e-09      4.73e-16    .0000519
-----------------------------+------------------------------------------------
                sd(Residual) |   79.95023   9.993861      62.57756    102.1459
------------------------------------------------------------------------------
LR test vs. linear regression: chibar2(01) =     0.00 Prob >= chibar2 = 1.0000
```

As mentioned before, we here use Wilcoxon's sum of rank to check robustness of the identified difference

```
. ranksum apd90, by(caco)

Two-sample Wilcoxon rank-sum (Mann-Whitney) test
```

```
        caco |      obs    rank sum    expected
-------------+---------------------------------
           1 |       21         231       346.5
           2 |       11         297       181.5
-------------+---------------------------------
    combined |       32         528         528

unadjusted variance      635.25
adjustment for ties       -0.12
                     ----------
adjusted variance        635.13

Ho: apd90(caco==1) = apd90(caco==2)
             z =  -4.583
    Prob > |z| =   0.0000
```

The robustness of the results is confirmed.

The statistical analysis demonstrated a significant difference of APD90 between control and HCM samples.

EXAMPLE 5: 50% decay time of Ca transients at 1Hz in human myocytes (Fig. 5E, left part).

Variable name= “Cat50”
caco=1 : control subjects
caco=2 : HCM subjects
subj : individual patients

- STEP 1) non-Gaussian distribution

The data were tested for normality using the Skewness/Kurtosis test

```
..sktest Cat50

                   Skewness/Kurtosis tests for Normality
                                                    ------- joint ------
    Variable |    Obs   Pr(Skewness)   Pr(Kurtosis)  adj chi2(2)    Prob>chi2
-------------+---------------------------------------------------------------
       Cat50 |     23      0.8667         0.2968         1.21         0.5458
```

Data is not normally distributed

- STEP 2) Heteroschedasticy (unequality of variances)

```
. sdtest Cat50, by(caco)

Variance ratio test
------------------------------------------------------------------------------
   Group |  Obs       Mean     Std. Err.   Std. Dev.   [95% Conf. Interval]
---------+--------------------------------------------------------------------
       1 |    15   222.0667    16.09036     62.3177    187.5563    256.5771
       2 |     8      279.5    15.10794    42.73172    243.7754    315.2246
---------+--------------------------------------------------------------------
combined |23    242.0435    12.91206    61.92406    215.2655    268.8214
```

```
------------------------------------------------------------------------------
    ratio = sd(1) / sd(2)                                f =   2.1268
Ho: ratio = 1                          degrees of freedom =    14, 7

    Ha: ratio < 1            Ha: ratio != 1           Ha: ratio > 1
  Pr(F < f) = 0.8404       2*Pr(F > f) = 0.3191        Pr(F > f) = 0.1596
```

Variances are equal

RESULTS OF STEP 1 AND 2: Although the variances in HCM and control samples were equal, overall datapoints are not normally distributed. Therefore, non-parametric test based on rank transformation (Wilcoxon's sum of rank and Kruskal-Wallis) will be used at the end to check robustness of results under violation of condition 1- or 2- .

- STEP 3) Within-subject correlation

We first tested whether inter-subject variability is higher that the variability among myocytes from the same subject, using One-Way ANOVA

```
.. loneway Cat50 subj
           One-way Analysis of Variance for Cat50:

                                    Number of obs =        23
                                        R-squared =    0.3838

    Source                SS          df       MS             F      Prob > F
-------------------------------------------------------------------------
Between subj          32376.957       7     4625.2795      1.33     0.3009
Within subj             51984        15     3465.6
-------------------------------------------------------------------------
Total                 84360.957      22     3834.5889

         Intraclass       Asy.
         correlation      S.E.        [95% Conf. Interval]
         ------------------------------------------------
           0.10525     0.24347       0.00000     0.58245

         Estimated SD of subj effect           20.1906
         Estimated SD within subj             58.86935
         Est. reliability of a subj mean      0.25073
              (evaluated at n=2.84)
```

Inter-subject variability is not significantly higher

Nonetheless, we use linear mixed models to calculate p values.

```
. . xtmixed Cat50 caco ||  subj:

Performing EM optimization:

Performing gradient-based optimization:

Iteration 0:   log likelihood =   -124.579
Iteration 1:   log likelihood = -124.39682
Iteration 2:   log likelihood = -124.39643
Iteration 3:   log likelihood = -124.39643
```

```
Computing standard errors:

Mixed-effects ML regression                     Number of obs      =        23
Group variable: subj                        Number of groups   =         8

                                        Obs per group: min =         2
                                                       avg =       2.9
                                                       max =         4


                                        Wald chi2(1)       =      5.89
Log likelihood = -124.39643                 Prob > chi2        =    0.0152

------------------------------------------------------------------------------
       Cat50 |      Coef.   Std. Err.      z    P>|z|     [95% Conf. Interval]
-------------+----------------------------------------------------------------
        caco |   57.43333   23.65568     2.43   0.015     11.06906    103.7976
       _cons |   164.6333   33.81586     4.87   0.000     98.35547    230.9112
------------------------------------------------------------------------------


------------------------------------------------------------------------------
  Random-effects Par|   Estimate   Std. Err.     [95% Conf. Interval]
-----------------------------+------------------------------------------------
subj: Identity               |
                  sd(_cons) |   1.45e-11    1.22e-10      1.04e-18   .0002022
-----------------------------+------------------------------------------------
               sd(Residual) |   54.03338   7.966816      40.47239    72.13821
------------------------------------------------------------------------------
LR test vs. linear regression: chibar2(01) =     0.00 Prob >= chibar2 = 1.0000
```

As mentioned before, we here use Wilcoxon's sum of rank to check robustness of the identified difference

```
. . ranksum Cat50, by(caco)

Two-sample Wilcoxon rank-sum (Mann-Whitney) test

        caco |      obs    rank sum    expected
-------------+---------------------------------
           1 |       15         145         180
           2 |        8         131          96
-------------+---------------------------------
    combined |23         276         276

unadjusted variance       240.00
adjustment for ties        -0.24
                      ----------
adjusted variance         239.76

Ho: Cat50(caco==1) = Cat50(caco==2)
             z =  -2.260
    Prob > |z| =   0.0238
```

The robustness of the results is confirmed.

The statistical analysis demonstrated a significant difference of Cat50 between control and HCM samples.
