# Supplementary material for: Slower Calcium Handling Balances Faster Cross-Bridge Cycling in Human MYBPC3 HCM
Source: Circ Res. 2023 Feb 6;132(5):628–44. doi: 10.1161/CIRCRESAHA.122.321956 (PMC9977265; doi:10.1161/CIRCRESAHA.122.321956)
Supplement: Supplementary file 2 [file res-132-628-s002.pdf]

Major Resources Table

In order to allow validation and replication of experiments, all essential research materials listed in the Methods should be included in the Major Resources Table below. Authors are encouraged to use public repositories for protocols, data, code, and other materials and provide persistent identifiers and/or links to repositories when available. Authors may add or delete rows as needed.

Animals (in vivo studies)

Species	Vendor or Source	Background Strain	Sex	Persistent ID / URL

Genetically Modified Animals

	Species	Vendor or Source	Background Strain	Other Information	Persistent ID / URL
**Parent - Male**					
**Parent - Female**					

Antibodies

Target antigen	Vendor or Source	Catalog #	Working concentration	Lot # (preferred but not required)	Persistent ID / URL
MyBP-C	Gift from Dr. Sakthivel Sadayappan		1:1000		
Secondary goat anti-rabbit horseradish peroxidase (HRP) conjugated antibody	GE Healthcare	RPN4301	1:1000		

DNA/cDNA Clones

Clone Name	Sequence	Source / Repository	Persistent ID / URL

Cultured Cells

Name	Vendor or Source	Sex (F, M, or unknown)	Persistent ID / URL
WTC11	Gift of the Regnier Lab	M	
ID3	Patient PBMC Reprogramming	M	
cID3	CRISPR-Cas9 editing of ID3	M	

Data & Code Availability

Description	Source / Repository	Persistent ID / URL

DOI [to be added]

PrimePCR Assay

Amplicon Context Sequence	Vendor /Source	Persistent ID and Catalog No
CACCTCCTCGTTGGCATTGACGTAGGTGGTGCGGAAGTTGATGAGGATG TCCAC AATGAACATGATGTCCACGATGAGGTCCACCACAGCCAGC	Bio-Rad Laboratories S.r.l.- Italy	KCNH2<br><br>UniGene ID Hs.647099<br><br>Unique Assay ID qHsaCED0037638
GCGCGGACGCTAGGCACACCAGCTTGGAGTTAGTTCAGCAAACCCCTTTT TCTT GCAGTGCGATTTCAGGTCTGGAACCTCAGCCTGTTGCTGACATATCAGGG CTGC CCGGGATAAAAGCAGAACATCTCGGATCCTATTCAAATGTCAAC	Bio-Rad Laboratories S.r.l.- Italy	KCND3<br>UniGene ID Hs.666367<br><br>Unique Assay ID qHsaCID0009820
ATCGCGGCAGCAACACGATCGGCGCCCGCCTGAACCGAGTAGAAGACA AGGTG ACGCAGCTGGACCAGAGGCTGGCACTCATCACCGACATGCTTCACC	Bio-Rad Laboratories S.r.l.- Italy	KCNQ1<br><br>UniGene ID Hs.95162<br><br>Unique Assay ID qHsaCID0007481
CTGGAAGAAGCTCTCCACGTGTTCCCTTGGGGCCTCCTCCCGGAGTGCAG GGT ACGTGTACTTGCCCATCATGTCATAGATGGACTTCATGATGTCAAGCATT	Bio-Rad Laboratories S.r.l.- Italy	KCNIP2<br><br>UniGene ID Hs.97044<br><br>Unique Assay ID qHsaCED0038529
CGGAAGGGCAGGAGGAGGATAGAGCCACAAGAGTGAGCACAGAAGTG ACAAGG GCAGAATCAGTGTGTGCTTGTGACAAGTATGGAAATGTCATGCCTTTAG GTTCAG TCCTATAAGGTAGGTGTATCAGTAAGGGCATTGATTCTGCGACCTTAACA GAGAT CAGAATAACAGT	Bio-Rad Laboratories S.r.l.- Italy	KCNE1<br><br>UniGene ID Hs.121495<br><br>Unique Assay ID qHsaCED0001056

DOI [to be added]

ARRIVE GUIDELINES

The ARRIVE guidelines (https://arriveguidelines.org/) are a checklist of recommendations to improve the reporting of research involving animals. Key elements of the study design should be included below to better enable readers to scrutinize the research adequately, evaluate its methodological rigor, and reproduce the methods or findings.

Study Design

**Groups**	**Sex**	**Age**	**Number (prior to experiment)**	**Number (after termination)**	**Littermates (Yes/No)**	**Other description**
Group 1 (Control)						
Group 2						
Add more if needed						

Sample Size: Please explain how the sample size was decided Please provide details of any a *prior* sample size calculation, if done.

Inclusion Criteria

Exclusion Criteria

Randomization

Blinding

DOI [to be added]
